# Reproductive ecology of the black rat (*Rattus rattus*) in Madagascar: the influence of density‐dependent and ‐independent effects

**DOI:** 10.1111/1749-4877.12750

**Published:** 2023-07-11

**Authors:** Kathryn SCOBIE, Soanandrasana RAHELINIRINA, Voahangy SOARIMALALA, Fehivola Mandanirina ANDRIAMIARIMANANA, Corinne RAHAINGOSOAMAMITIANA, Toky RANDRIAMORIA, Soloandry RAHAJANDRAIBE, Xavier LAMBIN, Minoarisoa RAJERISON, Sandra TELFER

**Affiliations:** ^1^ School of Biological Sciences University of Aberdeen Aberdeen UK; ^2^ Plague Unit, Institut Pasteur de Madagascar Antananarivo Madagascar; ^3^ Association Vahatra Antananarivo Madagascar

**Keywords:** Madagascar, *Rattus rattus*, reproductive ecology, rodent control

## Abstract

The black rat (*Rattus rattus*) poses a severe threat to food security and public health in Madagascar, where it is a major cause of pre‐ and post‐harvest crop losses and an important reservoir for many zoonotic diseases, including plague. Elsewhere, ecologically based rodent management (EBRM) strategies have been developed using ecological information to inform decisions on where and when to target control. EBRM could deliver improved health and well‐being outcomes in Madagascar if adapted to the local ecological context. Using data collected from removal studies, we explored spatio‐temporal patterns in the breeding activity of the black rat (*R. rattus*) in domestic and agricultural habitats across Madagascar and investigated to what extent these trends are influenced by rainfall and rat density. We identified clear spatio‐temporal variation in the seasonality of *R. rattus* reproduction. Reproduction was highly seasonal both inside and outside of houses, but seasonal trends varied between these two habitats. Seasonal trends were explained, in part, by variation in rainfall; however, the effect of rainfall on reproductive rates did itself vary by season and habitat type. A decline in breeding intensity with increasing rat density was recorded outside of houses. This has important implications for control, as populations may compensate for removal through increased reproduction. We recommend that sustained control initiated before the main breeding season, combined with improved hygiene and adequate rodent‐proofing in homes and grain stores, could curtail population growth and reduce pre‐ and post‐harvest losses provided that these measures overcome the compensatory response of rodent populations.

## Graphical abstract

Outside of houses, *Rattus rattus* reproduction increased during the rainy season and decreased during the dry season. Inside houses, reproduction continued year‐round but peaked at the onset of the dry season. A decline in breeding intensity with increasing rat density was recorded outside of houses. This has important implications for control as populations may compensate for removal through increased reproduction.

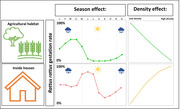

## INTRODUCTION

Globally, rodent pests pose a serious threat to food security and public health (Meerburg *et al.* 2009; Capizzi *et al.* 2014). Control efforts largely depend on the use of rodenticides and other lethal methods (Capizzi *et al.* 2014); however, owing to their behavioral plasticity, high mobility, and reproductive potential, rodent populations can compensate for removal through immigration (Krebs *et al.* 1978; Sullivan *et al.* 2001, 2003), increased survival (Brown & Tuan 2005), and better breeding performance (Singleton *et al.* 1999).

Ecologically based rodent management (EBRM) (Singleton *et al.* 1999) can provide environmental and economic benefits by limiting negative impacts of control on the environment and non‐target species and reducing reliance on chemical controls (Singleton *et al.* 2005, 2007; Brown *et al.* 2006). So far, EBRM has been successfully applied in agricultural settings, but its application within domestic settings could help to reduce post‐harvest losses (Belmain *et al.* 2003, 2015; Htwe *et al.* 2021) and lessen the risk of rodent‐borne disease (Lee *et al.* 2018).

The key to an EBRM approach is understanding the spatial and seasonal population dynamics of the target species, including density‐ and resource‐dependence of reproduction, and using this to inform decisions on when and where to target control. For example, in agricultural settings, implementing controls before the main breeding season and when population numbers are low can reduce rodent crop damage and subsequent yield losses compared with controls implemented arbitrarily or after detection of damage (Singleton *et al.* 2005; Brown *et al.* 2006; Huan *et al.* 2010; Palis *et al.* 2011; Stuart *et al.* 2020).

In Madagascar, non‐native small mammals are reservoirs of many zoonotic diseases, including the plague (Chanteau *et al.* 1998; Duplantier *et al.* 2005). Rodent pests also threaten food security. Surveys in Madagascar's eastern regions estimated that rodent pests cause average pre‐harvest losses of 53% of maize crops (range: 30–80%) and 38% of rice crops (20–100%), while post‐harvest losses were estimated at 11.5 kg (3–20 kg) and 13.3 kg (1.5–60 kg) per household per annum for maize and rice, respectively (Constant *et al.* 2020).

Madagascar's current strategy of rat management focuses on managing plague outbreaks, with an emphasis on using chemical insecticides to control the flea vector rather than the rat reservoir (Belmain *et al.* 2018); however, the occurrence of insecticide resistance is of major concern in the Madagascar's public health context (Miarinjara & Boyer 2016; Rajonhson *et al.* 2017). The public are also provided with information on non‐lethal rodent control measures (e.g. habitat management, locally made live capture traps) but uptake of this advice is limited (Belmain *et al.* 2018; Soarimalala *et al.* 2019; Constant *et al.* 2020). Instead, lethal control methods are more common (e.g. locally made snap‐traps, acute poisons) (Soarimalala *et al.* 2019; Constant *et al.* 2020). Whether these efforts are effective at reducing rodent numbers or rodent damage is unclear; however, studies elsewhere in sub‐Saharan Africa concluded that ad hoc control by smallholder farmers is largely ineffective, while inadequate and incorrect use of poisons fuels resistance (Belmain 2010).

EBRM has the potential to improve rodent pest management within Madagascar (Constant *et al.* 2020). Strategies must, however, be adapted to the local agricultural and ecological context (Singleton 1999). While research elsewhere has linked the breeding activity of rodent crop pests to the timing of agricultural cycles (e.g. Brown *et al.* 2005; Htwe *et al.* 2012; Stuart *et al.* 2015), our understanding of the reproductive cycles of rodent pests in agricultural and domestic habitat in Madagascar is based on a limited number of studies conducted at a small number of sites (Duplantier & Rakotondravony 1999). Moreover, the ecology and population dynamics of rodent pests will vary across Madagascar's heterogeneous landscape: Madagascar is a vast country encompassing arid, temperate, and tropical climates which, combined with complex topography, produce diverse vegetation types and ecosystems. Further modified by human activity, this diversity is mirrored in the agricultural landscapes which characterize the different bioclimate regions. Large rodent outbreaks are uncommon in Madagascar, but seasonal trends in abundance have been observed to vary with location, land‐use, and habitat type (Duplantier & Rakotondravony 1999). To be effective, EBRM programs must likewise reflect this diversity. With a view to informing the development of EBRM in Madagascar, we combined data collected by different studies (investigating rodent‐borne disease) to examine the breeding activity of the black rat, *Rattus rattus* (Linnaeus, 1758), in domestic and agricultural habitats across the country. Several key reproductive rates contribute to rodent fecundity, including sexual maturity (the physiological ability to reproduce), pregnancy rates, and litter size. We first explored seasonal patterns in these reproductive rates within different habitats and bioclimate regions, before investigating to what extent they are influenced by rainfall and rat density, and whether the effect of these processes on reproduction varies spatially and temporally. Based on our findings, we make recommendations for implementing EBRM strategies in Madagascar.

## MATERIALS AND METHODS

### Small mammal trapping

#### Study areas

Data used in this study were collected as part of three studies into rodent‐borne disease. The first study was conducted in the districts of Antsirabe (Latitude: 19.8659°S, Longitude: 47.0333°E) and Betafo (Lat: 19.8333°S, Long: 47.85°E) in Madagascar's Central Highlands. The region has a temperate climate with a cool–dry season (May–October) and warm–wet season (November–April). Small mammal trapping was conducted at 12 villages in Antsirabe and 12 in Betafo between January 2009 and February 2010 (one village per district sampled each month). Four of the villages were resampled a further four times between June 2010 and March 2011.

**Figure 1:**
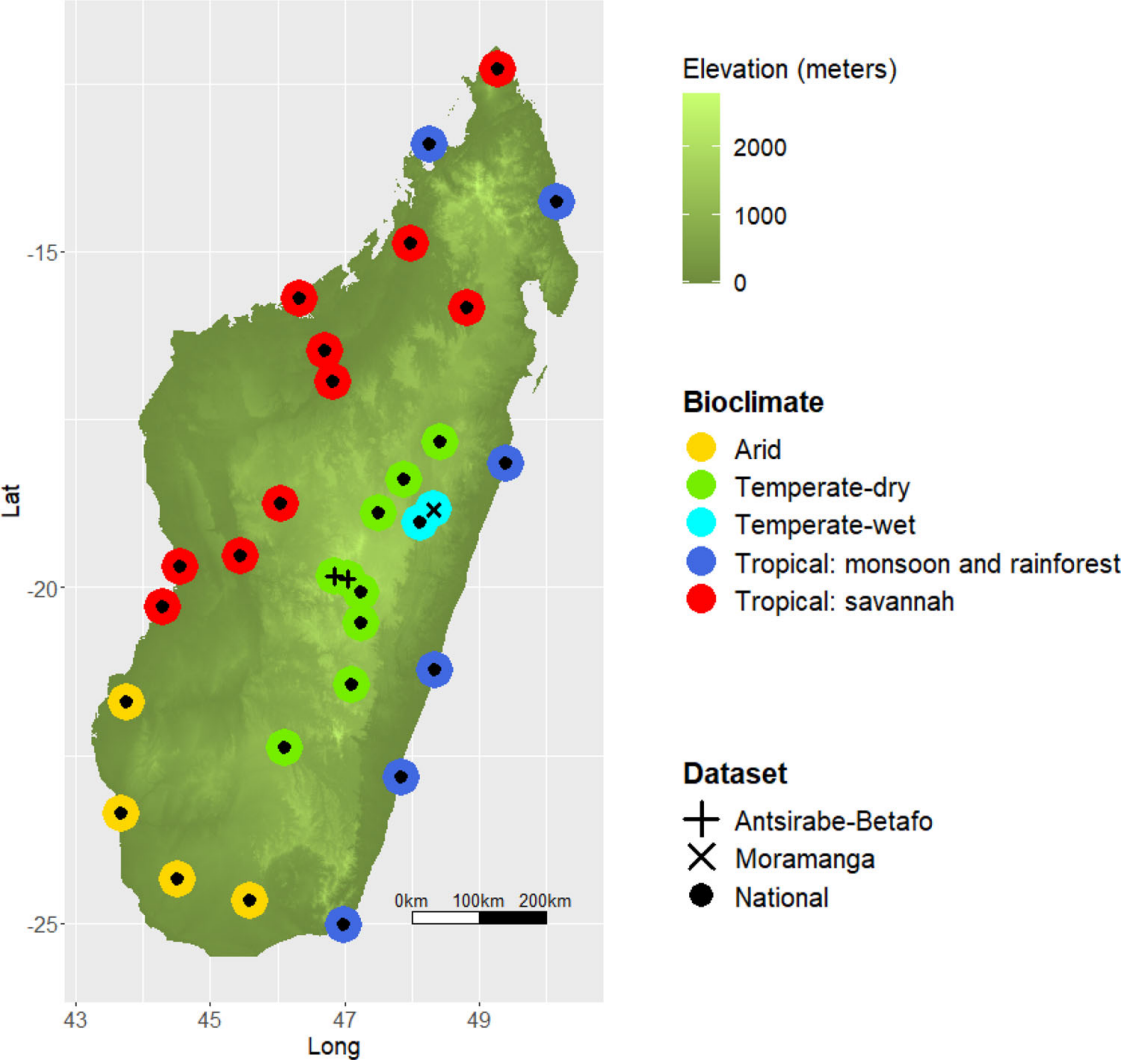
Map of sampling locations in the National (●), Antsirabe/Betafo (**+**), and Moramanga studies (**×**). Colored circles indicate local bioclimate: arid (*n* districts [sites] = 4 [8]), temperate‐wet (*n* = 1 [27]), temperate‐dry (*n* = 8 [38]), tropical rainforest (*n* = 6 [12]), and tropical savannah (*n* = 10 [20]).

In the second study, sampling was carried out at 28 districts across Madagascar (Fig. 1), encompassing arid, temperate, and tropical bioclimates (National study, herein). Sampling was carried out once per site during the dry season only (October–April) between 2011 and 2013.

The third study was conducted in the Moramanga district (Lat: 19.9495°S, Long: 48.2301°E), central eastern Madagascar, between 2013 and 2016. Moramanga's climate is temperate with no well‐defined dry season and hot–wet summers (December–April). Here, sampling was conducted at 20 village sites and five forest sites. Each site was sampled twice. Eleven of the sites were sampled first during the rainy season (December–April) and for a second time during the drier months (July–October). Fourteen of the sites were sampled first during the dry season and for a second time during a subsequent rainy season.

#### Sampling protocol

With permission from local authorities and household heads, live‐capture small mammal traps were set both inside houses and outside. We highlight that the trapping protocol followed in these studies was not implemented with the intention of rodent control but rather to sample the small mammal population.

Inside houses, all three studies followed the same protocol: one wire‐mesh BTS trap (30L × 10W × 10H cm, BTS Company, Besançon, France) and one Sherman trap (23L × 7.5W × 9H cm, H.B. Sherman Traps Inc., Tallahassee, Florida, USA) placed in areas thought to be used by rats but out of reach of children and non‐target species (4–32 houses/site, median = 17).

Outside of houses, three habitat types were sampled: (i) the private space immediately surrounding the exterior of the house (“household proximity”), (ii) areas of vegetation within approximately 150 m from houses (“village proximity”), and (iii) other habitats within 150–350 m from houses (“outside of village”) and including agricultural land, plantations, woodland, and forest (Table 1).

**Table 1:** Description of outdoor habitats sampled. In the Antsirabe/Betafo and National studies, habitats sampled outside of the village comprised irrigated rice and low‐lying agriculture typically within 150–300 m from the village. In the Moramanga study, habitats sampled outside of the village included a range of habitat types within approximately 350 m from the village

Antsirabe/Betafo	Moramanga	National	Habitat	Definition
Outside of village				
	x		Forest	Trees with closed‐canopy
	x		Savoka	Re‐generating forest and woodland
	x		Tanety	Hillside agriculture
x	x	x	Rice fields	Irrigated rice fields and low‐lying agriculture
	x		Plantation	Forested areas comprising plantations of pine or eucalyptus
Within village				
x		x	Village proximity	Vegetation within and bordering the village (dominated by sisal, bamboo, and bushes)
x	x		Household proximity	The private space immediately surrounding a house

Where such habitat was available, the household proximity was sampled using one BTS trap per house (1–19 houses/site, median = 5.5). The village proximity and habitat outside of the village were sampled using traplines comprising 10–20 Sherman, BTS, and/or wire‐mesh National traps (39.2L × 12.3W × 12.3H cm, Tomahawk Trap Company, Hazelhurst, Wisconsin, USA) positioned at 10–20 m intervals. Between 1 and 10 traplines were set at each site. All traps were baited either with dry fish and onion or raw manioc root and peanut butter and set for three (Antsirabe/Betafo and National studies) or five consecutive nights (Moramanga study). Traps were checked daily. No pre‐baiting took place.

Small mammal sampling in Moramanga included areas with natural habitat and was conducted under the following authorizations for research from the Madagascar Ministry of Environment and Forests: 154/13/MEF/SG/DGF/DCB.SAP/SCB, dated 3 July 2013; 312/13/MEF/SG/DGF/DCB.SAP/SCB (27 December 2013); 178/14/MEF/SG/DGF/DCB.SAP/SCB, (2 July 2014); and 11/15/MEF/SG/DGF/DCB.SAP/SCB (19 January 2015). Rodent handling was done in accordance with directive 2010/63/EU of the European Parliament (http://eur‐lex.europa.eu/Lex‐UriServ/LexUriServ.do?uri=OJ:L:2010:276:0033:0079:EN:PD) and the guidelines of the American Society of Mammalogists for the use of wild animals in research and education (Sikes *et al.* 2016).

#### Defining reproductive rates

Captured animals were humanely euthanized by cervical dislocation. Sex, weight, head–body length (HBL), and other morphometric measurements were recorded. Species identification was based on phenotypic characteristics.

Female reproductive status was determined using internal observations made at necroscopy. The following metrics were used as a proxy measure for population fecundity: proportion of females which were sexually mature (“female maturity”), proportion of sexually mature females which were pregnant (“gestation rate”), and litter size of pregnant females (number of visible embryos without magnification). We defined sexually mature females as those which were visibly pregnant or showed signs of previous pregnancy (uterine scars). Females below 45.0 g (the minimum weight at which an individual was found to be sexually mature) were excluded from the analysis.

### Statistical analysis

We used generalized linear mixed models (GLMMs) to examine the effect of rainfall and rat density on *R. rattus* reproduction and to explore spatio‐temporal variation in these effects. Female maturity and gestation rate were modeled using GLMMs with binomial errors and a logit link function. Litter size was modeled using generalized Poisson GLMMs with a log link function. The package glmmTMB (Brooks *et al.* 2017) in the software R (R Core Team 2020) was used to fit models.

As seasonal variation in reproduction has been observed between rats trapped inside and outside of houses (Duplantier & Rakotondravony 1999), to limit model complexity in‐house and external captures were modeled separately. To account for an expected effect of age on reproduction, models included an additive effect of centered and standardized HBL. HBL is correlated with age in wild *R. rattus* populations (Miller & Miller 1995) but is not influenced by reproductive state. Models also included a random effect of site and of sampling occasion (“mission”) nested within site.

#### Hypotheses and matching covariates

We first conducted an exploratory analysis of patterns related to season, bioclimate, and habitat. To explore whether seasonal trends vary between different bioclimate regions, global models included two‐way interactions between season and bioclimate region. Season was modeled using a sine and cosine term to test for cyclic seasonal patterns depending on date of capture, where date was converted to Julian day.

Spatial variation in climate (especially aridity) may influence primary productivity, and thus resource availability. To account for this potential variation, we considered four different bioclimate characterizations based on Köppen–Geiger climate classifications (Peel *et al.* 2007; Beck *et al.* 2018). The most complex of these (*Bio.5*) allowed seasonality in reproductive rates to vary across five bioclimate classes and was sensitive to differences in temperature and precipitation*. Bio.4* grouped temperate sites (warmest month ≥ 10°C and the coldest month 0–18°C). As rainfall may play an important role in driving reproduction, the third characterization (*Bio.3*) grouped tropical and arid sites (where the coldest month ≥ 18°C and/or mean annual temperature ≥ 18°C) but divided temperate sites (representing the largest proportion of our dataset) into those with dry winters (temperate–dry) and those with no dry season (temperate–wet). Finally, *Bio.2* differentiated between temperate sites and sites with hotter climates.

In external areas, resource availability may also be influenced by proximity to houses and agricultural fields. To account for this potential variation in our external analyses, and to explore whether the effect of habitat varies seasonally, global models included an effect of habitat type in a two‐way interaction with season. We initially considered two different habitat characterizations which grouped captures by their proximity to human habitation and agriculture: *Habitat* distinguished between habitat within the household proximity (the private space immediately surrounding the exterior of the house), the village proximity, and outside of the village. The second characterization, *Household.proximity*, distinguished between habitat within the household proximity and all other external habitat. In subsequent analyses investigating the effect of rat density on reproductive rates (see below), we removed data from the household proximity as low sampling effort (due to lower availability of suitable habitat in which to place traps) meant we had limited data to estimate rat density in these areas. In these analyses, habitat was therefore characterized as a 2‐level categorical variable, allowing reproductive rates to vary between habitat within and outside of the village proximity (*Within_village.vs.Outside_village*).

Following the exploratory analysis, we investigated the effects of rainfall and rat density on reproductive rates. Rainfall effects may vary seasonally as well as between different bioclimates and habitat types; therefore, global models included rainfall in two‐way interactions with season, bioclimate, and habitat. Eight rainfall characterizations were considered: total 30‐day rainfall with a 0–3 month lag (*Lag.n*) and total 90‐day accumulated rainfall with a 0–3 month lag (*Acc.lag.n*). District‐level precipitation data were obtained from the Climate Hazards group Infrared Precipitation with Stations (CHIRPS) dataset (Funk *et al.* 2015) and variables were centered and standardized. As described above, data from the household proximity were excluded from these analyses.

The effect of population density on reproductive rates may vary by season, habitat, and by age. To account for this potential variation, global models included density in two‐way interactions with season, habitat, and HBL. For both in‐house and external analyses, four indices of rat density were evaluated (eight total), reflecting total population density (including adults and subadults but excluding juveniles) and adult‐only population density at the site‐level and at the household‐ or trapline‐level. To estimate population density, abundance estimates were calculated in Program MARK (White & Burnham 1999; Supporting Information 1). For external captures, density per hectare was estimated at the site (*External.site*) and trapline‐level (*External*.*trapline*) by dividing the respective abundance estimates by the approximate area sampled. Using published estimates of *R. rattus* home ranges in Madagascar (Rasolozaka 1999; Ratsimanosika 1999) and forested areas of New Caledonia (Duron *et al.* 2020) and California (Whisson *et al.* 2007), this calculation assumed a 30‐m buffer around traps. For in‐house captures, abundance estimates calculated at the site‐level were corrected for the number of houses trapped and are thus expressed as the number of rats per house (*Household.site.level*). As small sample sizes meant that abundance estimates could not be calculated at the individual household‐level, household‐level capture rates were used as a measure of relative abundance per house (*Household.capture.rate*), calculated as the number of captures divided by the number of traps containing rodents or not sprung plus half the number of traps which were sprung but which had not caught a rodent (Theuerkauf *et al.* 2011).

To explore whether density effects varied between younger individuals potentially breeding for the first time and older individuals, we considered an interaction between density and HBL. Two characterizations of HBL were considered: a continuous variable (*Length.cont*) and categorical variable (*Length.cat*) which grouped individuals <150 mm in HBL and those ≥150 mm.

Definitions of levels included within the categorical explanatory variables of habitat type and bioclimate region are provided in Table 2, along with definitions of the different density and rainfall variables tested.

**Table 2:** Definition of habitat type, bioclimate region, density, and rainfall variables (or levels, for categorical variables) tested in generalized linear mixed model (GLMM) analysis of variables predicting the reproductive rates of female *Rattus rattus*. Bioclimate classifications are taken from Beck *et al.* (2018) and adapted from Peel *et al.* (2007). *MAP*,   mean annual precipitation (mm y^−1^); *MAT*, mean annual air temperature (°C). Precipitation threshold (*P*_threshold_) = 2 × *MAT* if >70% of precipitation falls in winter; 2 × MAT + 28 if >70% of precipitation falls in summer; or 2 × *MAT* + 14

Variable characterisation or level	Definition
Habitat type:	
Household proximity	The private space immediately surrounding the exterior of the house.
Village proximity	Vegetation within and bordering the village, up to ∼150 m from houses (dominated by sisal, bamboo, and bushes)
Outside of village	Other habitats within 150–350 m from houses and including agricultural land, plantations, woodland, and forest.
Bioclimate region:	
Temperate	Air temperature in the hottest month is >10°C; air temperature in the coldest month is >0°C and <18°C; *MAP* is ≥10 × *P*_threshold_
Temperate‐dry	As for temperate, but with dry winters and summers are hot or warm
Temperate‐wet	As for temperate, but with hot summers and no dry season
Tropical rainforest	Air temperature of the coldest month is ≥18°C; *MAP* is ≥10 × *P*_threshold_ and precipitation in the driest month is ≥60 mm
Tropical savannah	Air temperature of the coldest month is ≥18°C; *MAP* is ≥10 × *P*_threshold_ and precipitation in the driest month is <100‐*MAP*/25 and <60 mm
Arid	Mean annual temperature is ≥18°C and *MAP* is <10 and ≥5 × *P*_threshold_
Density:	
*External.site*	Total or adult‐only population density of *Rattus rattus* outside of houses (ha^–1^). Abundance estimates were generated at the site‐level and divided by the approximate area sampled.
*External.trapline*	Total or adult‐only population density of *R. rattus* outside of houses (ha^–1^). Abundance estimates were generated at the trapline‐level and divided by the approximate area sampled by each trapline.
*Household.site.level*	Total or adult‐only population density of *R. rattus* inside houses. Abundance estimates were generated at the site‐level and divided by the number of households sampled, generating a density estimate per household.
*Household.capture.rate*	Total or adult‐only capture rate of *R. rattus* inside houses.
Rainfall:	
*Lag.n*	Total rainfall over the previous 30 consecutive days prior to trapping with a lag of *n* (0–3) months
*Acc.lag.n*	Total rainfall over the previous 90 consecutive days prior to trapping with a lag of *n* (0–3) months

#### Model selection

We adopted an AIC‐based approach to covariate and model selection using Akaike's information criterion values corrected for small sample size (AICc) where models with an AICc value of within 2 of the lowest AICc (ΔAICc ≤ 2) were considered competitive (Burnham & Anderson 2002). First, the following global models (GM1 and GM2) were defined based on the a priori hypotheses outlined above:

**GM1**: Reproduction outside of houses ∼ (Rainfall * Season) + (Rainfall * Bioclimate) + (Rainfall * Habitat) + (Density * Season) + (Density * Habitat) + (Density * HBL)

**GM2**: Reproduction inside houses ∼ (*Rainfall* * *Season*) + (*Rainfall* * *Bioclimate*) + (*Density* * *Season*) + (*Density* * *HBL*)

Model selection was then carried out in three stages (a flow diagram outlining the process is provided in Supporting Information 2). In Stage 1, we identified which characterizations of the different explanatory variables to include in global models. Specifically, we evaluated characterizations of habitat, bioclimate, rainfall, and density when modeled in a two‐way interaction with season. Density characterizations were also modeled in a two‐way interaction with HBL. Rainfall characterizations appearing in models with ΔAICc ≤ 2 were also modeled in interaction with bioclimate. To reduce complexity, we considered each interaction separately. Simple additive models with no interactions were also considered. Separate models were thus run containing different characterizations of the same variable; these models were then compared using AICc. Characterizations which generated models with ΔAICc ≤ 2 were retained and used to construct a set of global models, each of which contained only a single characterization of each explanatory variable.

In Stage 2, we assessed simplifications of each global model using the “dredge” function in R's MuMIn package (Barton 2020), exploring all possible combinations of the variables of interest and generating a set of candidate models. Candidate models were subsequently grouped by response variable, ranked by AICc, and subset to include only those with ΔAICc ≤ 2 (“top model set” herein).

In Stage 3, we identified which model(s) from the top model set were most informative for interpretation. To avoid interpreting parameters which cannot be considered truly independent, models which included alternative characterizations of a variable included in a model with a lower AICc were excluded from the top model set. We also excluded models which were nested within the top model (Arnold 2010; Richards *et al.* 2011) and models that differed from other models with a lower AICc by the inclusion of a single parameter for which the 95% CIs overlap zero (Arnold 2010; Leroux 2019). Model validation was performed using R's DHARMa package (Hartig 2021). Residual and QQ‐plots for final models are provided in Figs S3, S4, Supporting Information 4, and Figs S5, S6, Supporting Information 5.

## RESULTS

Of 9670 *R. rattus* captured, 5105 were male, 4564 were female, and 1 was of unrecorded sex. Among females, 4280 (93.8%) were ≥45 g. Of these, 1284 (30.0%) were visibly sexually mature, 382 (29.8%) of which were pregnant. Median litter size was 6 both inside houses (IQR 4–7) and outside (IQR 5–7). A breakdown of captures per dataset, bioclimate, and habitat type is provided in Table 3 and Fig. S2, Supporting Information 3.

**Table 3:** Female captures (*n* and, in brackets, as a percentage of the total population) per dataset, bioclimate, and external habitat type, excludes juveniles (non‐reproductive individuals < 45 g). Mature refers to sexually mature females (pregnant or showing signs of previous pregnancy). Pregnant females were those with embryos at autopsy. Percentage of total is given in brackets. Litter refers to median (IQR) litter size (number of embryos at autopsy) of pregnant females

	Outside houses				Inside houses			
	Total	Mature	Pregnant	Litter	Total	Mature	Pregnant	Litter
*Dataset*								
Antsirabe‐Betafo	698	217 (31.1%)	43 (6.2%)	6 (5–7)	141	64 (45.4%)	22 (15.6%)	5 (4–6)
Moramanga	2028	637 (31.4%)	166 (8.2%)	6 (5–8)	178	92 (51.7%)	38 (21.3%)	6 (6–7)
National	271	106 (39.1%)	43 (15.9%)	6 (5–7)	339	168 (49.6%)	70 (20.6%)	5 (4–6.75)
*Bioclimate*								
Temperate‐dry (*n* = 27)	846	266 (31.4%)	65 (7.7%)	6 (5–7)	210	94 (44.8%)	35 (12.7%)	6 (4–7.5)
Temperate‐wet (*n* = 38)	2038	646 (31.7%)	170 (8.3%)	6 (5–7)	182	95 (52.2%)	41 (22.5%)	6 (6–7)
Tropical rainforest (*n* = 12)	28	18 (64.3%)	9 (32.1%)	5 (4–6)	66	36 (54.5%)	13 (19.7%)	5 (4–6)
Tropical savannah (*n* = 20)	46	21 (45.7%)	5 (10.9%)	6 (6–7)	133	68 (51.5%)	31 (23.3%)	5 (4–6)
Arid (*n* = 8)	39	9 (23.1%)	3 (7.7%)	4 (4–6)	67	31 (46.3%)	10 (14.9%)	5 (4–6.5)
*Habitat (external captures only)*								
House proximity	163	61 (37.4%)	22 (13.5%)	6 (5–6)				
Village proximity	421	134 (31.8%)	31 (7.4%)	6 (5–7)				
Outside of villages	2413	765 (31.7%)	199 (8.2%)	6 (5–7)				

Traps also captured the house mouse *Mus musculus* (in‐house: *n* = 742; outside: *n* = 287), the Asian shrew *Suncus murinus* (in‐house: *n* = 139; outside: *n* = 157), and *R. norvegicus* (in‐house: *n* = 91; outside: *n* = 124). The remainder of captures comprised native species, specifically Nesomyinae rodents and Tenrecidae tenrecs (in‐house: *n* = 4; outside: *n* = 156).

### Preliminary findings: season, bioclimate, and habitat effects

Our initial exploratory analysis explored patterns in reproduction related to season, habitat, and bioclimate. Full model selection results are provided in Supporting Information 4 (Tables S9, S10 for variable selection; Tables S11–S16 for top model sets; Tables S17, S18 for final models).

**Figure 2:**
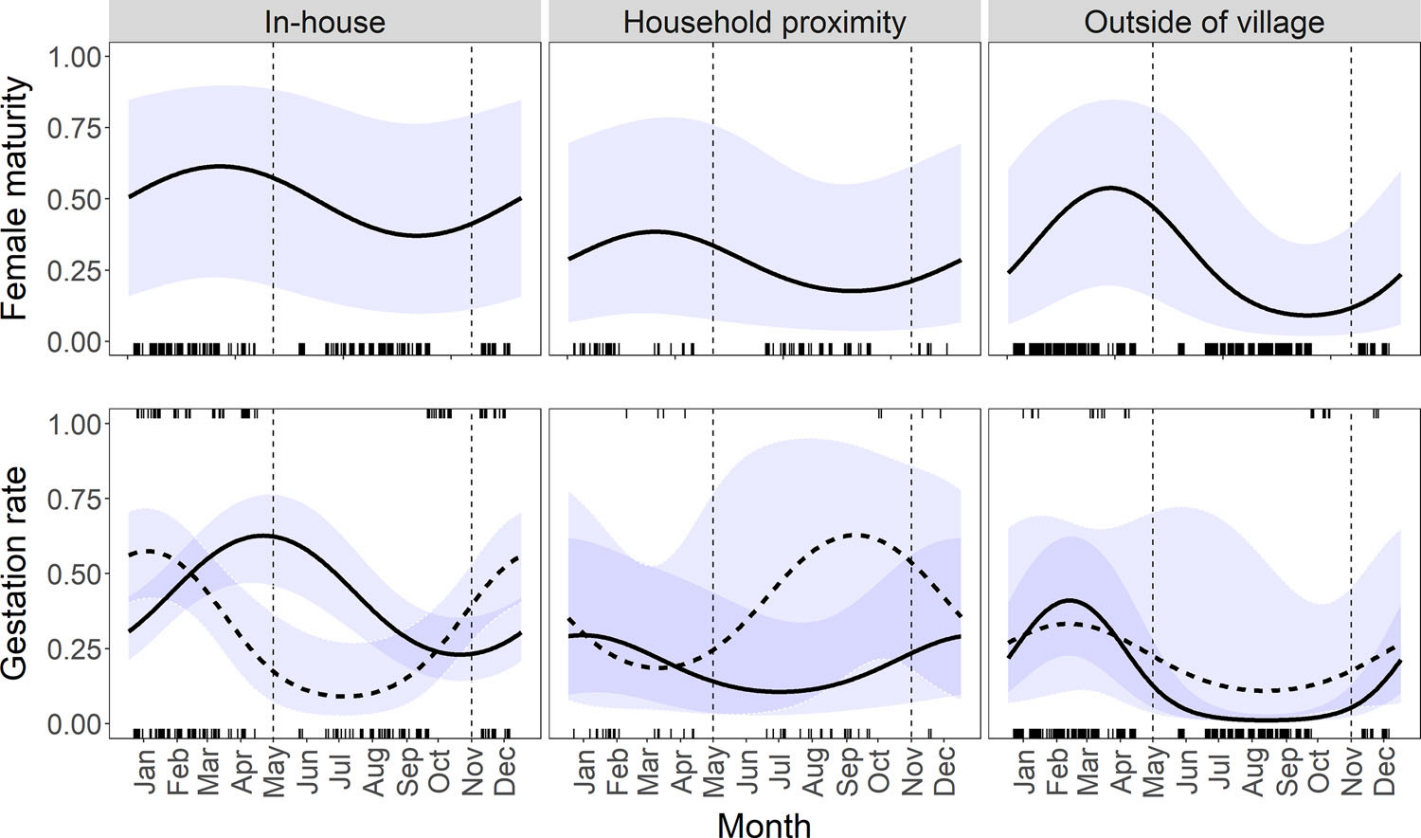
Predicted female maturity (above) and gestation rates (below) of *Rattus rattus* in different habitat types. Values (and 95% CI) are derived from binomial generalized linear mixed models (GLMMs) illustrating the effect of season and (for gestation rate) bioclimate region (temperate = solid line; non‐temperate = dotted line). Vertical dotted lines indicate the approximate start and end of the dry season (May–October). Tick marks indicate the distribution of observations within the full dataset (temperate regions at 0.0 on the *y*‐axis; non‐temperate regions at 1.0 on the *y*‐axis).

Results indicated that *R. rattus* reproduction was highly seasonal both inside houses and outside, typically increasing during the rainy season (November–April) and decreasing during the dry season (May–October) (Fig. 2). However, the timing and amplitude of the seasonal cycle varied between different habitats and bioclimates.

For female maturity, populations found outside of the village had the highest amplitude seasonal cycle; however, the timing of the seasonal cycle was similar across all habitat types (Fig. 2, top row). Additionally, the proportion of females which were sexually mature was highest inside houses year‐round. For gestation rates, seasonal patterns differed substantially between different habitat types and bioclimate regions (Fig. 2, bottom row). In particular, in temperate regions, gestation rates peaked in April–May inside houses, but peaked earlier outside of houses (December–January in the household proximity, February outside of villages). In contrast, in arid and tropical regions, the in‐house population appears to breed earlier or at a similar time compared to external populations. We cannot draw robust conclusions about differences between bioclimates in reproductive rates during the dry period due to limited sampling of non‐temperate regions between May and September. Female maturity and litter size also varied by bioclimate, primarily driven by low maturity and decreased litter size in arid sites, but we found no evidence that seasonal trends varied between bioclimates.

### Rainfall and density effects

#### Outside houses

Model selection produced one final model of female maturity and of gestation rate outside of houses, and two final models of litter size within 2 ΔAICc (Table 4). Models of female maturity and gestation rate included rainfall in two‐way interactions with habitat and season, with rainfall characterized as 90‐day accumulated rainfall lagged by 2 months (*Acc.lag.2*) and 3 months (*Acc.lag.3*), respectively. Both models also included bioclimate (*Bio.4*) as an additive effect (female maturity) or in interaction with rainfall (gestation rate), and population density as an additive effect (gestation rate) or in interaction with HBL (female maturity). The best models of gestation rate and litter size included HBL as an additive effect. The best models of litter size also included an additive effect of either season or of 90‐day rainfall with a 1‐month lag (*Acc.lag.1*). See Supporting Information 5 for full model selection results (Tables S19–S22 for variable selection; Tables S23–S25 for top model sets).

**Table 4:** Results of generalized linear mixed model (GLMM) analysis of variables predicting reproductive rates of female *Rattus rattus* outside of houses. Standardized regression coefficients (β) and standard error (SE) are presented for final models. Models included a random effect of site (Site) and mission nested within site (Site_mission : Site). Model parameters were included as additive effects or interactions (indicated by “x”). Variables included in final models were selected through an AIC‐based model selection process. For models of litter size, models 1 (m1) and 2 (m2) were considered competitive and so the results of both are presented. Reference levels: Bio.4 = Arid; Within_village.vs.Outside_village = Outside of village; Length.cat = ≥150 mm

						Litter size			
		Female maturity		Gestation rate		m1		m2	
Variable	Characterisation (level)	β	SE	β	SE	β	SE	β	SE
(Intercept)		−3.45	0.84	−3.01	1.86	1.58	0.05	1.61	0.04
Rainfall	*Acc.lag.1*	—	—	—	—	—	—	0.11	0.02
	*Acc.lag.2*	−0.71	0.4	—	—	—	—	—	—
	*Acc.lag.3*	—	—	−3.83	2.38	—	—	—	—
Season	*Season.sin*	1.73	0.52	1.64	0.32	0.24	0.05	—	—
	*Season.cos*	−0.76	0.36	1.29	0.48	0.02	0.05	—	—
Population density	*External.site* (Total)	−0.36	0.15	—	—	—	—	—	—
	*External.trapline* (Adult‐only)	—	—	−0.24	0.12	—	—	—	—
Bioclimate	*Bio.4* (Temperate)	1.96	0.81	1.16	1.85	—	—	—	—
	*Bio.4* (Tropical rainforest)	2.68	1.12	–50.9	24.52	—	—	—	—
	*Bio.4* (Tropical savannah)	2.36	1.04	–0.75	2.18	—	—	—	—
Habitat	*Within_village.vs.Outside_village* (Village prox.)	0.27	0.2	−0.09	0.28	—	—	—	—
Head–body length (HBL)	*Length.cont*	1.53	0.08	—	—	0.11	0.03	0.1	0.03
	*Length.cat* (<150 mm)	—	—	0.58	0.38	—	—	—	—
Rainfall × Season	Rainfall × *Season.sin*	1.03	0.28	0.63	0.31	—	—	—	—
	Rainfall × *Season.cos*	−0.4	0.27	0.3	0.32	—	—		
Rainfall × Habitat	Rainfall × *Within_village.vs.Outside_village* (Village prox.)	−0.49	0.18	0.59	0.3	—	—	—	—
Rainfall × Bioclimate	Rainfall × *Bio.4* (Temperate)	—	—	3.67	2.3	—	—	—	—
	Rainfall × *Bio.4* (Tropical rainforest)	—	—	−51.5	25.19	—	—	—	—
	Rainfall × *Bio.4* (Tropical savannah)	—	—	6.2	5.34	—	—	—	—
HBL × Population density	*Length.cont* × *External.site* (Total)	−0.3	0.08	—	—	—	—	—	—
**Random effect variance (SD)**									
Site_mission: Site		1.129 (1.067)		<0.001 (<0.001)		0.01 (0.1)		0.013 (0.112)	
Site		<0.001 (<0.001)		0.05 (0.22)		<0.001 (<0.001)		<0.001 (<0.001)	

**Figure 3:**
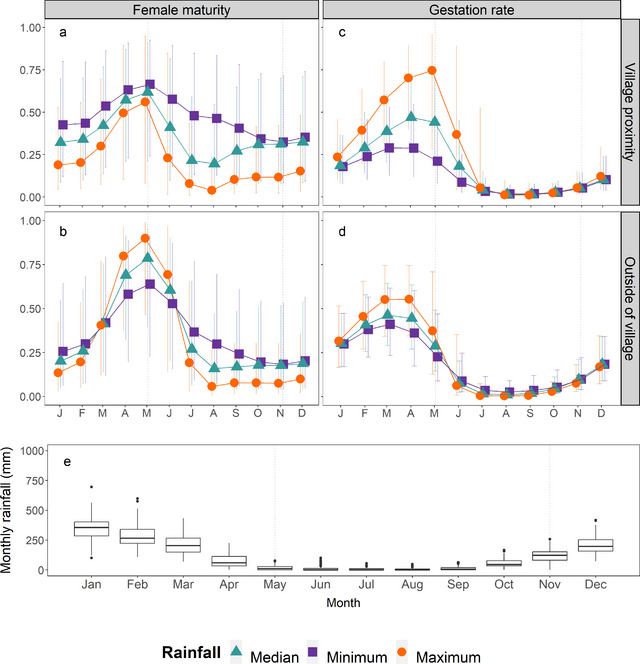
Predicted female maturity (a,b) and gestation rates (c,d) of *Rattus rattus* in external habitat. Values (and 95% CI) are derived from binomial generalized linear mixed models (GLMMs) illustrating the rainfall‐season interaction (comparing lines within the same plot) and rainfall‐habitat interaction (comparing plots a,b and c,d). Rainfall was characterized as 90‐day rainfall with a 2‐month (*Acc.lag.2*, female maturity) or 3‐month lag (*Acc.lag.3*, gestation rate). Predictions are based on the minimum, median, and maximum *Acc.lag.2* and *Acc.lag.3* values recorded at temperate sites between 2008 and 2020. Date of capture = 1st of each month; bioclimate region = temperate. (e) Monthly rainfall recorded at temperate sites between 2008 and 2020. Vertical dotted lines indicate approximate start and end of the dry season (May–October).

The effect of lagged rainfall on maturity and gestation rates varied seasonally and between different habitat types. Figure 3 illustrates these effects in temperate regions. In the village proximity, female maturity was negatively associated with *Acc.lag.2* (Fig. 3a). Outside of the village, however, there was a positive association between *Acc.lag.2* and female maturity between April and June (when female maturity was highest), corresponding to increased rates of maturity with increased November–February rainfall (Fig. 3b).

Gestation rates showed a positive association with *Acc.lag.3* rainfall during the main breeding season (April–June), corresponding to October–January rainfall (Fig. 3c,d). This trend was most pronounced within the village proximity (Fig. 3c), where predicted gestation rates for May range from 21.1% (7.8–45.8%) at the lowest recorded *Acc.lag.3* rainfall (282.3 mm) to 74.6% (27.7–95.7%) at the highest (1100.0 mm). During the rest of the year, gestation rates remained low irrespective of previous rainfall.

The additive effect of bioclimate on female maturity appeared to be driven by a relatively low proportion of sexually mature females in arid bioclimates (Table 4). Meanwhile, the relationship between rainfall and gestation rates varied between different bioclimates. In particular, gestation rates at tropical rainforest sites were negatively associated with Acc.lag.3 (odds ratio for a 10‐unit increase in Acc.lag.3 = 0.14 [0.02–0.82]). We also found evidence that seasonality in gestation rates shifted within these bioclimates, peaking earlier in the year than at temperate and tropical‐rainforest sites; however, our sample of sexually mature females from non‐temperate regions was small (*n* = 39), and so this relationship requires further investigation.

**Figure 4:**
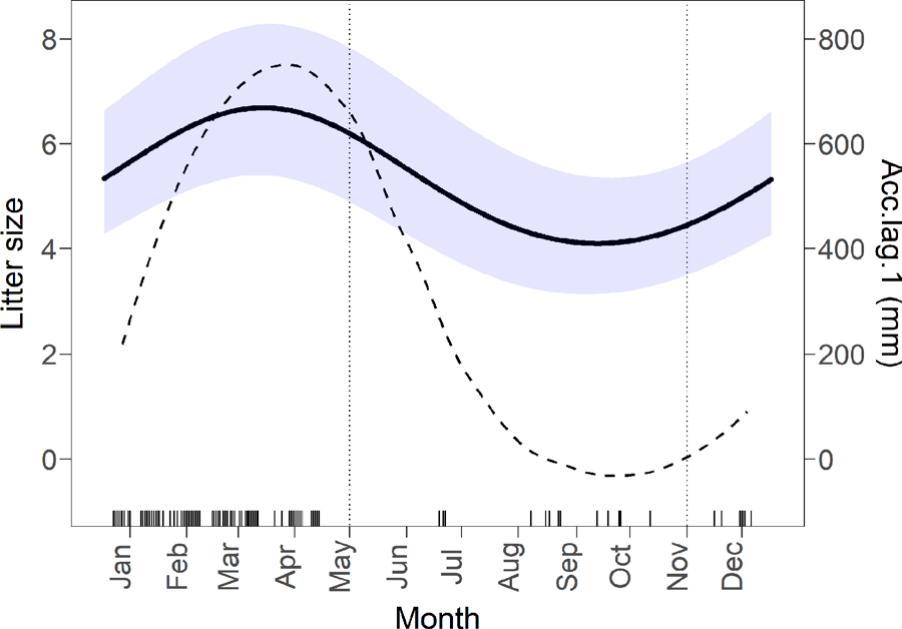
Predicted litter size of *Rattus rattus* in habitat outside of houses (solid line). Values (and 95% CI) derived from binomial generalized linear mixed model (GLMM) illustrating the effect of season (litter size model “m1” in Table 4). Head–body length (HBL) is set to the median value. Dashed line indicates median 90‐day rainfall with a 1‐month lag (Acc.lag.1) recorded across all study sites between 2008 and 2020. Vertical dotted lines indicate the approximate start and end of the dry season (May–October). Tick marks indicate the distribution of observations within the full dataset.

Litter size increased throughout the rainy season to a peak in April, coinciding with the main breeding season, and decreased during the dry season when gestation rates were low (Fig. 4). We found no evidence that seasonality varied between bioclimates or habitat types, or that it was modified by the influence of rainfall. However, the seasonal pattern did mirror seasonal fluctuations in 90‐day rainfall with a 1‐month lag (*Acc.lag.1*); notably, in the second model within top model set for litter size, season was replaced by an additive effect of *Acc.lag.1* rainfall (ΔAICc = 1.4), suggesting that seasonal variation in litter size may be driven by variation in rainfall.

Density estimates ranged from 0 to 45.3 individuals/hectare (or 0–25.5 adults/hectare), recorded in January and July, respectively (median: 10.8 inds/ha and 7.4 adults/ha). An increase in adult population density was associated with a decrease in gestation rates among sexually mature individuals (odds ratio 0.96 [0.92–1.00]). This relationship was not found to be influenced by season, habitat, or HBL.

**Figure 5:**
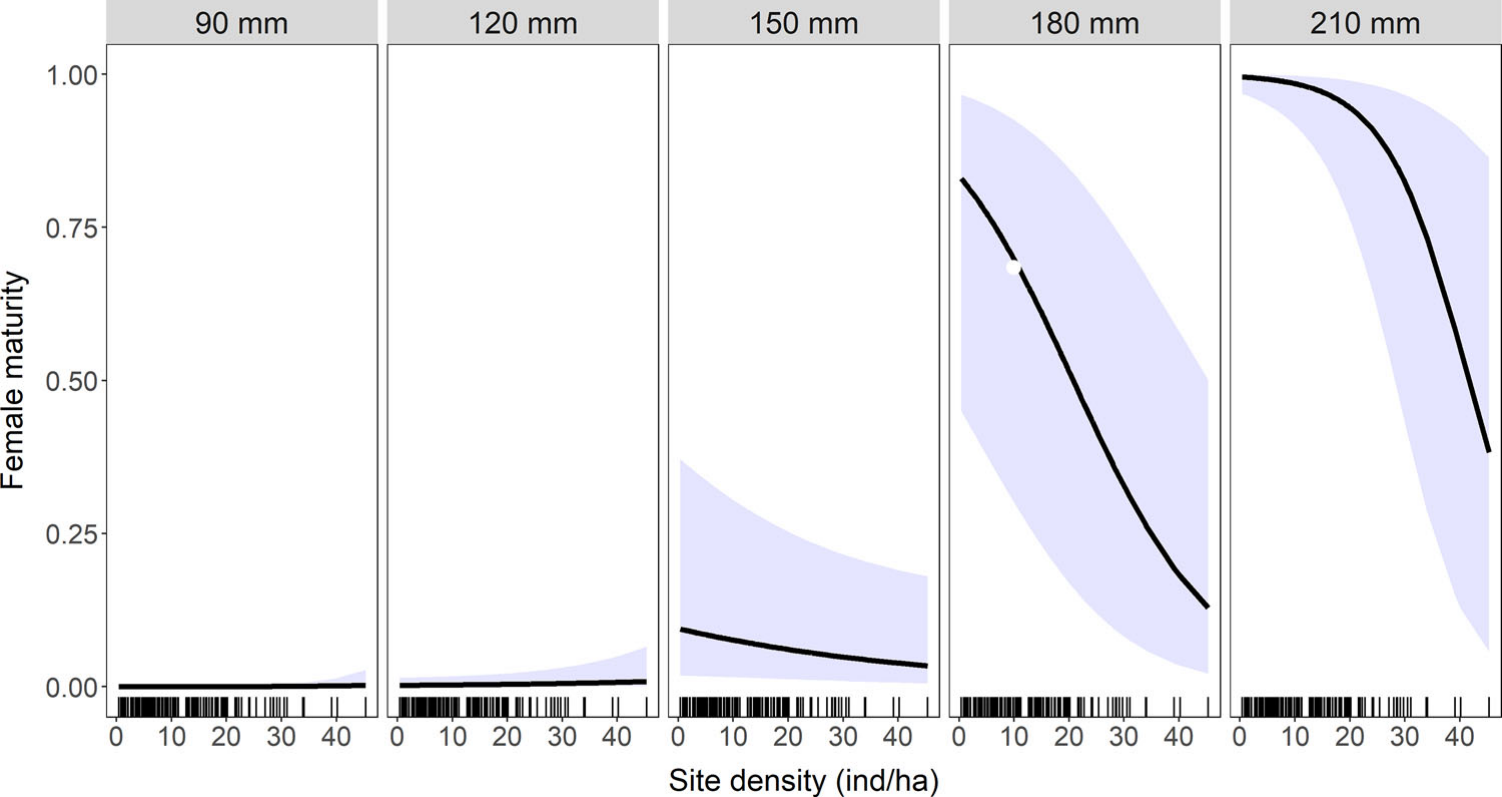
Predicted maturity of female *Rattus rattus* in habitat outside of houses. Values (and 95% CI) are derived from binomial generalized linear mixed model (GLMM) illustrating the interaction effect of total population density and head–body length (HBL). Tick marks indicate the distribution of observations within the full dataset.

Meanwhile, an increase in total population density was associated with a decrease in female maturity, though this relationship was modified by HBL. The HBL of females ≥ 45g captured outside of houses ranged from 92 to 210 mm (median: 156 mm). Using HBL as a proxy for age, our results indicate that population density has little effect on the probability that a young individual (i.e. 90–150 mm) will reach sexual maturity (Fig. 5). As individuals age, they are more likely to have reached sexual maturity, though the effect of density‐dependent breeding suppression also increases. As such at high population densities, the probability that a 210‐mm female has reached sexual maturity remains as low as 38.3% (5.7 –86.4%).

The best model predicted a 7.0% increase in litter size for every 10‐mm increase in HBL (3.6–10.6%). Conversely, the best model of gestation rate found that individuals <150 mm in HBL had a higher probability of being pregnant than larger individuals (odds ratio 1.78 [0.85–3.73]). It is not clear from our data whether this effect represents genuine senescence or a bias in the gestation dataset, with younger individuals having a lower probability of having completed their first pregnancy.

#### Inside houses

Model selection produced one final model for each of the three reproductive rates inside houses (Table 5). The best model of female maturity included an additive effect of 90‐day rainfall with a 1‐month lag (*Acc.lag.1*) and a two‐way interaction between HBL (*Length.cont*) and total population density estimated at the site‐level (*Household.site.level*). The best model of gestation rate included an effect of 30‐day rainfall with a 3‐month lag (*Lag.3*) in a two‐way interaction with season, and an additive effect of *Household.site.level*. The best model of litter size included an additive effect of bioclimate (*Bio.2*), 30‐day rainfall with a 1‐month lag (*Lag.1*), and *Length.cont*. Full model selection results are given in Supporting Information 5 (Tables S26–S28 for top model sets).

**Table 5:** Results of GLMM analysis of variables predicting reproductive rates of female *Rattus rattus* inside houses. Standardized regression coefficients (β) and standard error (SE) are presented for final models. Models included a random effect of site (Site) and mission nested within site (Site_mission : Site). Model parameters were included as additive effects or interactions (indicated by ‘×’). Variables included in final models were selected through an AIC‐based model selection process. For models of litter size, models 1 (m1) and 2 (m2) were considered competitive and so the results of both are presented. *Bio.2* reference level = Arid and tropical

		Female maturity		Gestation rate		Litter size	
Variable	Characterisation (level)	β	SE	β	SE	β	SE
(Intercept)		−0.17	0.16	−0.43	0.27	1.62	0.05
Rainfall	*Acc.lag.1*	0.34	0.14	—	—	—	—
	*Lag.3*	—	—	0.69	0.38	—	—
	*Lag.1*	—	—	—	—	0.06	0.03
Season	*Season.sin*	—	—	0.04	0.37	—	—
	*Season.cos*	—	—	0.43	0.29	—	—
Population density	*External.site* (Total)	−0.42	0.16	0.39	0.15	—	—
Bioclimate	*Bio.2* (Temperate)	—	—	—	—	0.12	0.06
Head‐body length (HBL)	*Length.cont*	1.37	0.13	—	—	0.08	0.04
Rainfall × Season	*Lag.3* × *Season.sin*	—	—	−0.45	0.38	—	—
	*Lag.3* × *Season.cos*	—	—	−0.71	0.341	—	—
HBL × Population density	Length.cont × *Household.site.level* (Total)	0.4	0.17	—	—	—	—
**Random effect variance (SD)**							
Site_mission : Site		0.29 (0.54)		0.06 (0.23)		<0.001 (<0.001)	
Site		0.52 (0.72)		<0.001 (<0.001)		<0.001 (<0.001)	

**Figure 6:**
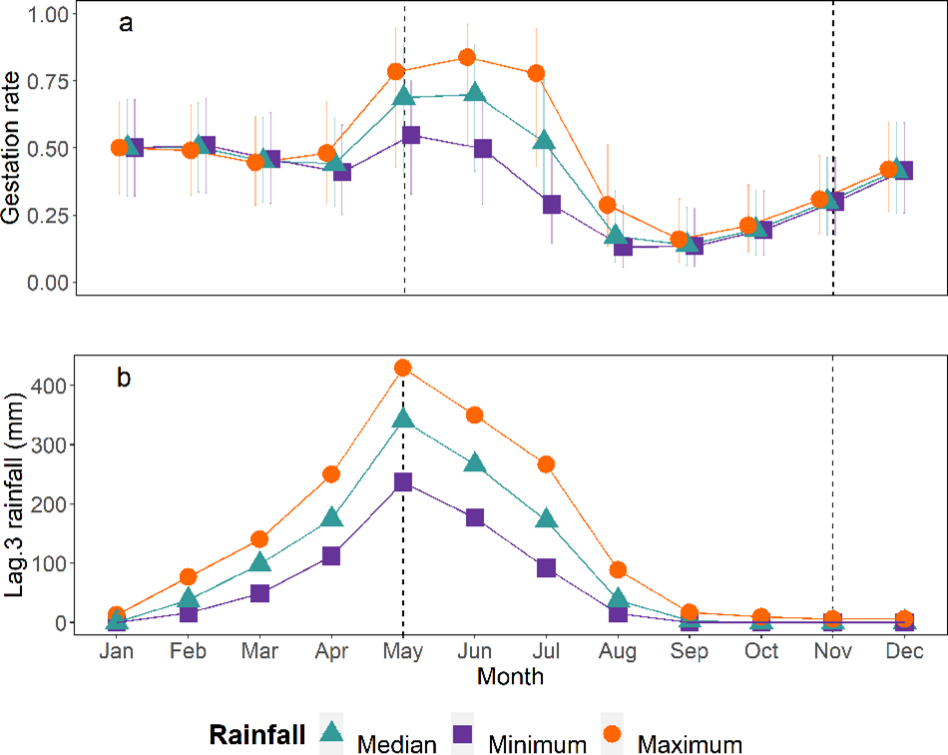
Predicted gestation rates of sexually mature *Rattus rattus* inside houses (a). Values (and 95% CI) are derived from binomial GLMM illustrating the interaction effect of season and 30‐day rainfall lagged by 3 months (Lag.3). Date of capture was specified as the 1st of each month. Predictions are based on the 75% quartile (maximum), median, and 25% quartile (minimum) Lag.3 rainfall recorded across all study sites between 2008 and 2020 (b). Vertical dotted lines indicate the approximate start and end of the dry season (May–October).

A 10‐mm increase in *Acc.lag.1* rainfall was predicted to increase the proportion of females which were sexually mature by 1.2% (0.3–2.2%). This effect was not modified by the influence of season or bioclimate. Between May and July, high *Lag.3* rainfall (corresponding to December–March rainfall) was also associated with an increase in gestation rate (Fig. 6a). We found no evidence that this effect varied between different bioclimates. These results indicate that the perceived seasonal variation in female maturity inside houses was driven by seasonal rainfall variation, and that while this lagged rainfall contributes to seasonal variation in gestation rates this relationship itself varies seasonally.

When rainfall effects were incorporated, models of litter size inside houses no longer indicated an effect of season. Instead, litter size was predicted to increase by 5.0% (1.0–9.0%) for every 100‐mm increase in *Lag.1* rainfall. This effect did not vary by season or bioclimate and suggests that apparent seasonal trends may be driven by seasonal variation in rainfall. Litter size was also increased by 13.0% (0.0–28.0%) at temperate sites compared with arid and tropical regions; however, our sample size from non‐temperate regions was small (*n* = 53).

**Figure 7:**
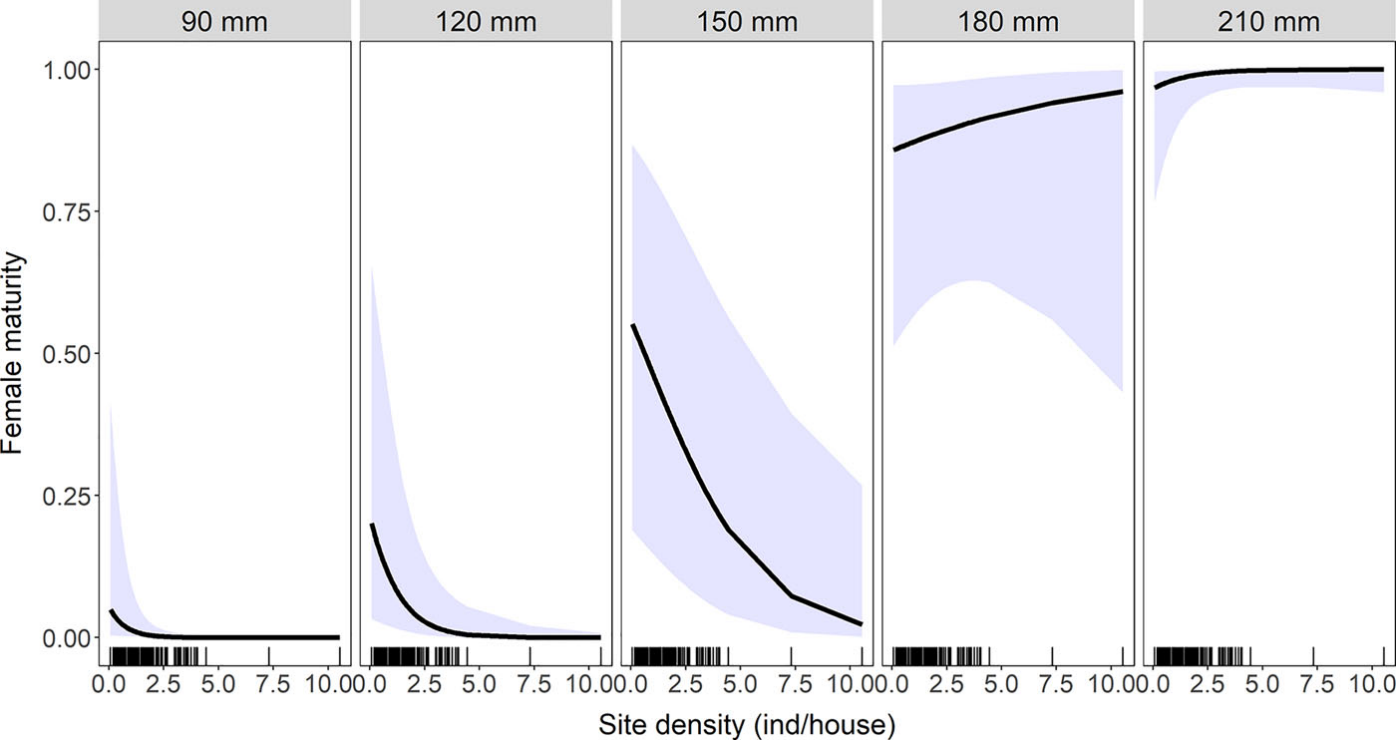
Predicted female maturity of female *Rattus rattus* inside houses. Values (and 95% CI) are derived from binomial generalized linear mixed model (GLMM) illustrating the interaction effect of total population density and head–body length (HBL). Tick marks indicate the distribution of observations within the full dataset (across all values of HBL).

Both female maturity and gestation rates were influenced by in‐house population density measured at the site level, which was estimated to range from 0 to 11 inds/house (median = 2 inds/house). For female maturity, the effect of population density was again modified by HBL (Fig. 7). In particular, at 120–170 mm, the probability of reaching sexual maturity was predicted to increase with increasing HBL but with a negative effect of population density. The nature of this interaction differed substantially compared to that observed for external populations and at low population densities, females inside of houses are able to reach sexual maturity at a younger age than those living outside of houses (compare Fig. 5 with Fig. 7).

Meanwhile, we found a positive association between population density and gestation rate which was modified by neither season nor HBL (Table 5). Litter size was positively associated with HBL, increasing by 5.3% (0.6–10.3%) for every 10‐mm increase in HBL.

## DISCUSSION

The reproductive rates of female *R. rattus* showed a clear seasonal cycle, the timing and amplitude of which varied between different habitats and bioclimate regions. Seasonal variation in rainfall contributed to seasonal patterns; however, the effect of rainfall on female maturity and gestation rates did itself vary seasonally and between different habitats. We also recorded a decline in breeding intensity with increasing rat density outside of houses, whereas high population densities were associated with increased reproduction inside houses. Outside, females living at low density were more likely to be sexually mature and more likely to be pregnant than if living at high density, especially those which were mid‐sized. Inside houses, however, gestation rates were higher among sexually mature females living at high population densities than those living at low densities.

An association between rainfall and reproduction in rodent crop pests is often attributed to the influence of rainfall on primary productivity (Clark 1980; Leirs *et al.* 1997; Sluydts *et al.* 2007; Previtali *et al.* 2009; Andreo *et al.* 2009b). We found that, in temperate regions of Madagascar, female reproductive rates outside of houses increased during the rainy season and decreased during the dry season, coinciding with the timing of the agricultural calendar. Additionally, increased rainfall during the early rainy season (November–January) was associated with an increase in female maturity and gestation rates during the main breeding season (April–May), particularly in habitats outside of villages and predominantly comprising agricultural land. Madagascar's main growing season begins in November–January (with the onset of the rainy season) and lasts for several months (Rigden *et al.* 2022). Our results therefore suggest that high rainfall at the start of the growing season, when crops are establishing, may provide optimal conditions for rat reproduction later in the rainy season.

During the dry season, a decline in reproductive rates coincided with the rice harvest, when the crop is brought into villages to be threshed and dried before being stored in granaries or houses or sold. At this time, gestation rates are predicted to fall to 0.0–3.0% outside of houses, irrespective of previous rainfall. Therefore, the cessation of reproduction during the dry season may be linked to the timing of the agricultural calendar. This would be consistent with observations from agricultural regions of Vietnam (Brown *et al.* 2005) and the Philippines (Htwe *et al.* 2012; Stuart *et al.* 2015), where the breeding activity of *Rattus* species was associated with crop stage.

Initiating rodent controls ahead of the main growing season is recommended to reduce pre‐harvest losses in agricultural settings, assuming that this also precedes the species’ main breeding season (Brown *et al.* 2005, 2006; Palis *et al.* 2011; Htwe *et al.* 2012). Our findings support this assumption for Madagascar, with reproductive rates peaking during the main growing season. Accordingly, we recommend that rodent controls are initiated in habitat outside of houses at the end of the dry season (before reproductive rates increase and before crops are planted). However, to deliver a net reduction in density, control programs must also overcome the density‐dependent response of the target species (e.g. Melero *et al.* 2015). We found that female maturity and gestation rates outside of houses increased at lower densities. Additionally, the effect of density on gestation rates was not influenced by age, suggesting that even large, sexually mature females tend not to breed at high densities. Due to these density‐dependent effects, reducing population density via removal risks expediting female maturation and increasing gestation rates. However, if these density effects are linked to changes in per capita resource availability (e.g. Adler 1998; Lewellen & Vessey 1998; Zhang *et al.* 2003; Andreo *et al.* 2009a), then reducing the carrying capacity of the environment (e.g. through rat‐proofing, improved hygiene) could mitigate local compensatory responses to control.

Inside houses, high and relatively consistent resource availability throughout the year can support year‐round reproduction in commensal rodents (Pocock *et al.* 2004; Gomez *et al.* 2008). Still, inconsistent resource availability in the domestic environment can trigger changes in demographic rates (Panti‐May *et al.* 2012). Indeed, we found that rat reproduction continued year‐round inside houses but was nonetheless highly seasonal. Here, gestation rates peaked between May and July, coinciding with the post‐harvest period. Just as masting events can trigger increases in rodent populations (Aplin & Lalsiamliana 2010; Belmain *et al.* 2010; Douangboupha *et al.* 2010; Jaksic & Lima 2003; Htwe *et al.* 2010), our finding indicates that in‐house populations can respond rapidly to resource fluctuations and increase reproduction when food availability spikes. As was found in the uplands of Lao PDR (Khamphoukeo *et al.* 2006), increases in the in‐house rodent population following the harvest have been attributed to the resource‐driven movement of rodents from fields to houses in agricultural regions of Madagascar (Rasolozaka 1999; Rahelinirina *et al.* 2010); however, our result shows that enhanced reproduction is also contributing to this effect.

Gestation rates inside houses were also influenced by rainfall. Specifically, increased rainfall during the rainy season (December–March) was associated with higher gestation rates between May and July. This suggests there is connectivity between in‐house rodent populations and external conditions, which may be attributable to the impact of rainfall on primary productivity and crop growth: a more successful harvest may lead to an increase in food availability when crops are brought into houses for storage, providing optimal conditions for rodents to reproduce. Alternatively, the relationship between rainfall and in‐house gestation rates could reflect functional connectivity and the exposure to external conditions of rodents that move into houses following the harvest (Rasolozaka 1999; Khamphoukeo *et al.* 2006; Rahelinirina *et al.* 2010).

As well as season and rainfall effects, we found that females reached sexual maturity at a younger age inside houses compared with outside, particularly at low population densities. This has implications for control, as removing individuals from the household population may expedite maturation among any remaining young females. Conversely, sexually mature females living inside houses were more likely to reproduce when population density was high compared to when population density was low. There are few documented examples of positive density dependence in wild rodent populations, which should theoretically occur only at small population sizes (Morris 2002) such as when low mate encounters inhibit reproduction (Courchamp *et al.* 1999). This is unlikely to be the case in this system due to the connectedness of in‐house and external populations (Brouat *et al.* 2013). Instead, increased reproduction at high population densities may reflect high resource abundance. Previous studies show substantial village‐to‐village variation in in‐house rodent abundance (Rahelinirina *et al.* 2021); villages with high rodent abundance inside houses may have more resources in and around houses than other villages, supporting reproduction even at high population densities. Further work is needed to explore relationships between reproductive rates and density inside houses, particularly in the context of active rodent management.

Although less attention has been paid to the application of EBRM strategies within domestic settings, our results reinforce findings from Bangladesh and Myanmar that controls initiated ahead of the main breeding season and before harvested crops are brought in for storage could curtail reproduction and reduce post‐harvest losses (Belmain *et al.* 2015). Improving hygiene around the home and rat‐proofing stored food could help manage the in‐house population in Madagascar by reducing environmental carrying capacity and preventing bursts in reproductive activity post‐harvest. However, the connectivity of in‐house and external rat populations (Brouat *et al.* 2013) will necessitate collective action among community members to reduce rodent access to resources.

### Management implications: not a one‐size‐fits‐all approach

In Madagascar's temperate regions, the proposed measures would equate to initiating rodent control in September–October outside of houses and in March–April inside houses. However, rainfall patterns and agricultural systems vary considerably across Madagascar, contributing to spatial variation in season and rainfall effects. In particular, we found evidence that seasonality and the effects of rainfall on gestation rates outside of houses varied between different bioclimate regions. While this study was only able to consider broad habitat categories, female reproductive rates and their seasonality may also vary between different outside habitat types (e.g. forest vs. non‐forest). This highlights a fundamental aspect of EBRM: that control strategies must be specific to the local ecological setting (Singleton *et al.* 1999). In Madagascar, this necessitates an understanding of the local agricultural and ecological context, at a fine scale, when designing control strategies.

Where rodents are reservoirs of zoonotic diseases, control risks increasing disease prevalence through impacts on movement and contact rates (e.g. Donnelly *et al.* 2006; Bielby *et al.* 2016; Lee *et al.* 2018; Smith & Delahay 2018). Therefore, studies must also evaluate how the proposed management strategies will impact on the movement of rodents and the distribution and abundance of disease vectors and infected rodents (Rahelinirina *et al.* 2021).

### Conclusions

In Madagascar, attempts to manage rodent pests generally depend on traps and poisons (Soarimalala *et al.* 2019; Constant *et al.* 2020). However, their application is typically reactive, implemented in response to unacceptable levels of damage or rodent numbers. Based on our findings, we recommend that future research should investigate the impact of sustained rodent control initiated before the main breeding season, combined with improved hygiene and adequate rodent‐proofing inside homes, on population growth and on pre‐ and post‐harvest losses in Madagascar. Additionally, we identified considerable spatial variation in the seasonality of *R. rattus* reproduction which must be reflected in the timing of control measures. Finally, research is needed to identify the plague risk associated with the proposed strategies. Until then, program managers must evaluate the local plague risk, balancing this against the risk of no control, and consider how control strategies are communicated to the general population to ensure that protocols are implemented correctly and safely.

## Supporting information


**Table S1** Habitat variables evaluated in trapline‐level Huggins’ p and c models of the capture probability of *Rattus rattus*.
**Table S2** Results of Huggins’ p and c models for *Rattus rattus* caught inside houses, ranked by AICc.
**Table S3** Results of Huggins’ p and c models for *Rattus rattus* caught in outside habitat, ranked by AICc.
**Table S4** Results of trapline‐level Huggins’ p and c models for adult *Rattus rattus* caught in outside habitat (National dataset only), ranked by AICc.
**Table S5** Results of trapline‐level Huggins’ p and c models for adult *Rattus rattus* caught in outside habitat (Antsirabe/Betafo dataset only), ranked by AICc.
**Table S6** Results of trapline‐level Huggins’ p and c models for adult *Rattus rattus* caught in outside habitat (Moramanga dataset only), ranked by AICc.
**Table S7** Results of trapline‐level Huggins’ p and c models for sub‐adult *Rattus rattus* caught in outside habitat, grouped by dataset and ranked by AICc.

**Figure S1** Flow diagram illustrating the three‐stage process followed during model selection.
**Figure S2** Capture rate of female *Rattus rattus* (≥45 g) grouped by (a) dataset and bioclimate and (b) month and habitat type.
**Figure S3** (a–d) QQ‐plot (left) and plot of standardized residuals versus model predictions (right) simulated from the fitted models of *Rattus rattus* reproductive rates outside of houses (Table S17). Red lines indicate quantile deviations detected.
**Figure S4** (a–d) QQ‐plot (left) and plot of standardized residuals vs model predictions (right) simulated from the fitted models of *Rattus rattus* reproductive rates inside houses (Table S18). Red lines indicate quantile deviations detected.
**Figure S5** (a–d) QQ‐plot (left) and plot of standardized residuals vs model predictions (right) simulated from the fitted models of *Rattus rattus* reproductive rates outside of houses (Table 3, main text). Red lines indicate quantile deviations detected.
**Figure S6** (a–c) QQ‐plot (left) and plot of standardized residuals vs model predictions (right) simulated from the fitted models of *Rattus rattus* reproductive rates inside houses (Table 4, main text). Red lines indicate quantile deviations detected.

**Table S9–Table S18** Results of GLMM analysis of variables predicting reproductive rates of female *Rattus rattus* inside houses.

**Table S19–Table S28** AICc values and standardized regression coefficients from GLMM analysis of variables predicting litter size of pregnant *Rattus rattus* inside houses, where rainfall and population density were included as independent variables in global models.

Supporting Information
