# Supplementary material for: Reproductive ecology of the black rat (Rattus rattus) in Madagascar: the influence of density‐dependent and ‐independent effects
Source: Integr Zool. 2023 Jul 11;19(1):66–86. doi: 10.1111/1749-4877.12750 (PMC10952345; doi:10.1111/1749-4877.12750)
Supplement: Supplementary file 1 — Table S1 Habitat variables evaluated in trapline‐level Huggins’ p and c models of the capture probability of Rattus rattus. Table S2 Results of Huggins’ p and c models for Rattus rattus caught inside houses, ranked by AICc. Table S3 Results of Huggins’ p and c models for Rattus rattus caught in outside habitat, ranked by AICc. Table S4 Results of trapline‐level Huggins’ p and c models for adult Rattus rattus caught in outside habitat (National dataset only), ranked by AICc. Table S5 Results of trapline‐level Huggins’ p and c models for adult Rattus rattus caught in outside habitat (Antsirabe/Betafo dataset only), ranked by AICc. Table S6 Results of trapline‐level Huggins’ p and c models for adult Rattus rattus caught in outside habitat (Moramanga dataset only), ranked by AICc. Table S7 Results of trapline‐level Huggins’ p and c models for sub‐adult Rattus rattus caught in outside habitat, grouped by dataset and ranked by AICc. [file INZ2-19-66-s002.docx]

**Supplementary Materials –** **Modelling capture probability**

Program MARK 9.0 (White & Burnham, 1999) was used to estimate abundance while modelling capture probability of *Rattus rattus*. The Huggins’ closed capture p and c model was selected as it allows for the use of individual covariates to model capture probability. Closed capture models were considered appropriate for a removal study and rodent populations were considered to be geographically and demographically closed during the 3-5 day trapping period.

To maximise power, we first combined data from all three datasets (Antsirabe/Betafo, Moramanga, and National study) to assess how capture probabilities varied between day of capture, season, sex and age. *R. rattus* were grouped into four age classes based on weight (females - class 1: <40 g, class 2: 40-79 g, class 3: 80-119 g, class 4: >120 g; males – class 1: <45 g, class 2: 45-89 g, class 3: 90-134 g, class 4: >135 g). Initial exploration indicated that the capture probability for age class 1 animals was low. This may reflect the ability of small-size individuals to escape from wire mesh cages, and/or the small home-range and short-distance movements of young. As our sample size was also low for this age class (in-house: n = 223 [12.7%]; outside: n = 270 [3.4%]), to avoid over-estimating their population size these individuals were excluded from subsequent analyses.

In-house and outdoor capture histories were modelled separately as we expected movement and interaction with traps to be very different within houses, and so we wanted to reduce the necessity to consider complex models with multiple interactions. As some sites were trapped more than once, data were grouped by both site and visit to generate site-level population size estimates for each visit. As we were interested in generating separate population size estimates for adults (age class 3-4) and sub-adults (age class 2), data were also grouped according to these two age categories. Models were specified as removal models, with recapture probability (*c*) fixed to 0 (Lukacs, 2018). The number of trapping occasions was specified as five (i.e., five consecutive days of sampling per site visit). To accommodate joint modelling with sites where trapping was only conducted for three days, we fixed capture probability (*p*) to 0 for days 4 and 5 when sampling did not occur (Cooch & White, 2018). By specifying capture probability as 0.0 for days 4 and 5 for the Antsirabe/Betafo and National studies, the model will only estimate the capture probability for days 1-3 for these datasets. The capture history for each individual thus consisted of five trapping occasions, followed by numerically (0/1) coded dummy variables corresponding to the site, visit, and age category (sub-adult or adult). Sex and age class were included as individual covariates.

#### Model selection philosophy

We adopted an information-theoretic approach to variable and model selection using Akaike’s Information Criterion values corrected for small sample size (AICc) (Burnham & Anderson, 2002). To avoid fitting overly complex models, modelling was conducted in two stages. Stage 1 began with a set of candidate models that included the following variables hypothesised to represent sources of potential variation in capture probability: day of capture (i.e., trapping occasion), season, sampling protocol, sex and age-class (detailed below). In Stage 1, candidate models included a maximum of two of these independent variables as additive or interaction effects where they were biologically meaningful.

In Stage 2, variables and/or interactions identified in Stage 1 as sources of variation in capture probability (i.e., those which reduced AIC by >2 compared to an intercept-only model and which, in the case of interactions, improved model fit compared to the equivalent additive model) were combined. Following a backwards selection approach, variables and/or two-way interactions were iteratively removed in order to identify the most appropriate model for the data based on AICc. We considered models with the lowest AICc value to be the best performing (relative to other candidate models), but models with an AICc value of within 2 of the lowest AICc (ΔAICc ≤2) were also considered competitive (referred to herein as the “top model set”).

To identify which model(s) within the top model set were most informative for interpretation, we excluded any which included alternative characterisations of a variable included in a model with a lower AICc or which were nested within the top model (Arnold, 2010; Richards *et al.,* 2011). Following the rationale of Arnold (2010) and Leroux (2019), we also excluded models from the top set that differed from other models with a lower AICc only by the inclusion of a single non-informative parameter (i.e., 95% CIs for the parameter overlap zero).

#### Variables considered

To test for a novel-object response, day of capture was modelled as a two-level categorical variable and AICc was used to compare two different groupings: *Day.1* grouped trapping occasions 2-5 together thus allowing capture probability to vary only on the first day of trapping, whilst *Day.2* grouped trapping occasions 1-2 and 3-5.

We defined seasons as either rainy or dry according to the amount of rainfall (mm) at the site during the 30 days prior to trapping using data obtained from the CHIRPS (Climate Hazards group Infrared Precipitation with Stations) dataset (Funk *et al.* 2015). We evaluated three different rainfall thresholds, above which sites were considered to be in their rainy season: 75 mm, 50 mm and 25 mm. Based on a comparison of AICc values, 50 mm was selected as the threshold.

To test the effect of sampling protocol on capture probability, dataset was modelled as a three-level categorical variable (*Dataset.3*) allowing capture probability to vary across the three datasets, and as a two-level categorical variable (*Dataset.2*) which grouped the Antsirabe/Betafo and National studies (which had very similar protocols, including the same bait type).

To explore possible intrinsic sources of variation in capture probability, we included sex and age as individual covariates. Sex was thus modelled as a binary (1/0 [male/female]) variable, whilst two different age classifications were considered: *Age.3* was a three-level categorical variable with age classes 2, 3 and 4 as levels (*Age.3*), and *Age.2* was a two-level categorical variable in which age classes 3 and 4 were grouped. For models of in-house trapping, we also tested whether the presence of other small mammal species within the house influenced *R. rattus* capture probability as, given the limited number of traps available within houses, interspecific competition for traps may lead to trap saturation. The presence of other species was thus included as an individual covariate (1/0 [present/absent]). The final top model sets are presented below (Tables S2-S3).

#### Trapline-level models

We have so far described in-house and outdoor trapping modelled at the site-level. For outdoor trapping, we also wanted to calculate estimates of abundance at the trapline-level and further assess whether capture probability was influenced by the local habitat of traplines. Additional analyses were therefore conducted in which data were grouped by trapline and visit. To provide estimates of abundance in the immediate vicinity of houses, individuals trapped in the house proximity were included for these analyses but were grouped by site and visit.

In order to handle the large number of traplines (or groups) within the MARK software, each of the three datasets were analysed separately. For this analysis, we also conducted separate analyses for adults and sub-adults in order to generate separate population size estimates for each. However, the low number of sub-adults captured in the National study (n = 33) meant that even simple additive models could not be estimated; we therefore did not conduct trapline-level analysis of the sub-adult population for this dataset.

We used the best models from the site-level analysis (where datasets were combined) as a starting point for model parameterisation, as the former have greater power to detect variation in capture probability than a reduced dataset. However, as the datasets were now to be analysed separately and individuals split by age group, dataset and age variables (i.e., *Dataset.2*, *Age.2* and *Age.3*) were not included in the models. For adult individuals, we therefore began with the following model: p(*Season* × *Sex* + *Day.1* × *Sex* + *Age3vs4*), where *Age3vs4* is a two-level categorical variable with levels corresponding to age class 3 and age class 4.

For the sub-adult population, we first re-evaluated whether the interaction effects identified in the combined dataset analysis were supported by the data; this was because most sub-adults were not breeding and so capture probabilities were not expected to be strongly influenced by behaviours linked to reproductive activity. Moreover, datasets were smaller for this category of individuals, resulting in problems estimating parameters for more complex models. Subsequently, we began with the following model for the Antsirabe/Betafo and Moramanga datasets: p(*Season* + *Day.1* + *Sex*).

For analyses of both adults and sub-adults, we then added in variables related to habitat, using a forward selection process to identify the most appropriate model for the data. Below we describe the habitat variables considered in each analysis (summarised in Table S1).

In the Antsirabe/Betafo dataset, we compared three different ways of modelling habitat type: *Habitat.3* was a three-level categorical variable allowing capture probability to vary across the three habitat types sampled, whilst *Rice.fields* and *House.prox.AB* were two-level categorical variables allowing capture probability to vary in rice fields and in the household proximity respectively.

In the Moramanga dataset, a preliminary analysis identified that, in terms of capture probability, forest transects within village sites were more similar to non-forest transects within village sites than transects in forest sites. In the adult population models, we subsequently compared four different ways of modelling habitat type, each of which included “Forest sites” as one distinct level: *Habitat.7* allowed capture probability to vary between all six habitat types within village sites and forest sites. In *Habitat.4,* transects within natural forest fragments or secondary forest (Savoka) were grouped (i.e., habitat typically characterised by a complex three-dimensional structure of trees and scrub/understorey, herein referred to as natural habitat) and transects within agricultural habitat (i.e., Tanety, Rice field, and Plantation) were grouped. *Natural.habitat* grouped agricultural habitat and habitat in the household proximity (i.e., human-altered habitat), and *House.prox.MORA* grouped natural and agricultural habitat, allowing capture probability to vary within the household proximity. For the sub-adult population models, our sample size in the household proximity was low (n = 68). Therefore, further exploration of habitat was restricted to the three-level *Natural.habitat* for this age group.

Finally, in the National dataset, habitat was modelled as a two-level categorical variable (*Habitat.2*), with capture probability allowed to vary between rice field habitat and habitat within the village proximity.

We ran models with habitat included as an additive effect and, for models of the adult population, in a two-way interaction with day of capture, season, age and sex. Interaction effects were not considered in models of the sub-adult population due to the lower sample size. The final top model sets are presented below (Tables S4-S7).

**Table S1** Habitat variables evaluated in trapline-level Huggins’ p and c models of the capture probability of Rattus rattus. The three datasets were modelled separately. There is some variation between the three datasets in the definition and type of habitats sampled, and thus in the habitat variables and levels tested.

| **Habitat variable** | **Levels** | | | | | | | | | | |
| --- | --- | --- | --- | --- | --- | --- | --- | --- | --- | --- | --- |
| Antsirabe/Betafo dataset | | | | | | | | | | | |
| *Habitat.3* | Rice fields | | | Village proximity | | | | | Household proximity | | |
| *Rice.fields* | Rice fields | | | Village and Household proximity | | | | | | | |
| *House.prox.AB* | Rice fields and Village proximity | | | | | | | Household proximity | | | |
| Moramanga dataset | | | | | | | | | | | |
| *Habitat.7* | Forest site | Forest transects | Savoka | | Tanety | | Rice field | | | Plantation | Household proximity |
| *Habitat.4* | Forest site | Natural | | | Agricultural habitat | | | | | | Household proximity |
| *Natural.habitat* | Forest site | Natural | | | Human | | | | | | |
| *House.prox.MORA* | Forest site | Natural and Agricultural habitat | | | | | | | | | Household proximity |
| National dataset | | | | | | | | | | | |
| *Habitat.2* | Rice fields | | | | | Village proximity | | | | | |

#### Top model sets

**Table S2** Results of Huggins’ p and c models for Rattus rattus caught inside houses, ranked by AICc. The top model set is presented together with corresponding model-specific beta coefficient estimates (± SE) for covariates determining capture probability (p). K = number of parameters, ΔAICc = difference in AICc value from top model. The following covariates were included as additive (+) or interaction (x) effects: day of capture (Day.1, capture probability varies on first day of trapping), dataset (Dataset.2, capture probability varies in the Moramanga study), age of rat (Age.3, capture probability varies between age classes 2, 3, and 4; and Age.2, capture probability varies for age class 2), and the presence of other rodent species (Other.sp). Reference values: Dataset.2 = Moramanga ; Age.2 = adult ; Age.3 = age class 4 ; Day.1 = days 2-5 ; Other.sp = absent. n captures = 1,520; n sites = 132.

|  |  |  |  | **Beta coefficients** | | | | | | |
| --- | --- | --- | --- | --- | --- | --- | --- | --- | --- | --- |
| **Model description** | **K** | **ΔAICc** | **Intercept** | ***Dataset.2*** | ***Age.2*** | ***Day.1*** | ***Other.sp*** | ***Age.3* (class 2)** | ***Age.3* (class 3)** | ***Dataset.2* x**  ***Age.2*** |
| p(*Dataset.2* + *Age.2*) | 3 | 0.00 | -0.78 ± 0.14 | -1.10 ± 0.34 | -1.11 ± 0.5 | - | - | - | - | - |
| p(*Dataset.2* + *Age.2* + *Day.1*) | 4 | 1.28 | -0.95 ± 0.26 | -1.11 ± 0.40 | -1.38 ± 0.83 | 0.09 ± 0.1 | - | - | - | - |
| p(*Dataset.2* + *Age.2* + *Other.sp*) | 4 | 1.30 | -0.74 ± 0.15 | -1.0 ± 0.35 | -1.04 ± 0.47 | - | -0.27 ± 0.34 | - | - | - |
| p(*Dataset.2* x *Age.2*) | 4 | 1.96 | -0.78 ± 0.14 | -1.12 ± 0.35 | -1.15 ± 0.55 | - | - | - | - | 0.29 ± 1.28 |
| p(*Dataset.2* + *Age.3*) | 4 | 1.98 | -0.76 ± 0.21 | -1.10 ± 0.34 | - | - | - | -1.13 ± 0.53 | -0.04 ± 0.27 | - |

**Table S3** Results of Huggins’ p and c models for Rattus rattus caught in outside habitat, ranked by AICc. The top model set is presented together with corresponding model-specific beta coefficient estimates (± SE) for the following covariates: dataset (Dataset.2, capture probability varies in the Moramanga study), age of the rat (Age.3, capture probability varies between age classes 2, 3, and 4; and Age.2, capture probability varies for age class 2), day of capture (Day.1, capture probability varying on first day of trapping), Sex of the rat (Sex), and Season (Season). Covariates were included as additive (+) or interaction (x) effects. K = number of parameters, ΔAICc = difference in AICc value from top model. Reference values: Dataset.2 = Moramanga; Age.2 = adult; Age.3 = age class 4; Day.1 = days 2-5; Sex = female; Season = dry. n captures = 7,657; n sites = 144.

|  |  |  |  |  | **Beta coefficients** | | | | | | | | | | | | |
| --- | --- | --- | --- | --- | --- | --- | --- | --- | --- | --- | --- | --- | --- | --- | --- | --- | --- |
| **Model description** |  | **K** | **ΔAICc** | **Int.** | ***Dataset.2*** | | ***Age.3* (class 2)** | | ***Age.3* (class 3)** | | ***Season*** | ***Day.1*** | | ***Sex*** | | ***Age.2*** | |
| p(*Dataset.2* x *Age.3* + *Dataset.2* x *Season* + *Dataset.2* x *Day.1* + *Day.1* x *Sex* +  *Season* x *Sex* + *Day.1* x *Age.2*) | | 14 | 0.00 | -1.18 ± 0.28 | -0.78 ± 0.32 | | -0.72 ± 0.42 | | -0.13 ± 0.22 | | 0.4 ± 0.22 | 0.17 ± 0.09 | | 0.44 ± 0.18 | | -0.72 ±0.42 | |
| p(*Dataset.2* x *Age.3* + *Dataset.2* x *Season* + *Dataset.2* x *Day.1* + *Day.1* x *Sex* +  *Season* x *Sex*) | | 13 | 0.47 | -1.11 ± 0.26 | -0.77 ± 0.31 | | -1.15 ± 0.45 | | -0.13 ± 0.21 | | 0.4 ± 0.22 | 0.13 ± 0.08 | | 0.4 ± 0.17 | | - | |
| p(*Dataset.2* x *Age.3* + *Dataset.2* x *Season* + *Day.1* x *Sex* + *Season* x *Sex* +  *Day.1* x *Age.2*) | | 13 | 0.70 | -1.00 ± 0.23 | -1.06 ± 0.25 | | -0.6 ± 0.33 | | -0.11 ± 0.2 | | 0.41 ± 0.2 | 0.07 ± 0.07 | | 0.48 ± 0.18 | | -0.6 ± 0.33 | |
|  |  |  | ***Dataset.2* x  *Age.3* (class 2)** | | | ***Dataset.2* x  *Age.3* (class 3)** | | ***Dataset.2* x  *Season*** | | ***Dataset.2* x  *Day.1*** | | | ***Day.1* x  *Sex*** | | ***Season* x  *Sex*** | | ***Day.1* x  *Age.2*** |
| p(*Dataset.2* x *Age.3* + *Dataset.2* x *Season* + *Dataset.2* x *Day.1* + *Day.1* x *Sex* +  *Season* x *Sex* + *Day.1* x *Age.2*) | |  | 0.55 ± 0.41 | | | 0.65 ± 0.27 | | 0.52 ± 0.24 | | -0.15 ± 0.09 | | | -0.17 ± 0.08 | | -1.0 ± 0.2 | | -0.14 ± 0.09 |
| p(*Dataset.2* x *Age.3* + *Dataset.2* x *Season* + *Dataset.2* x *Day.1* + *Day.1* x *Sex* + *Season* x *Sex*) | |  | 0.77 ± 0.5 | | | 0.63 ± 0.26 | | 0.49 ± 0.23 | | -0.16 ± 0.09 | | | -0.17 ± 0.07 | | -0.96 ± 0.19 | | - |
| p(*Dataset.2* x *Age.3* + *Dataset.2* x *Season* + *Day.1* x *Sex* + *Season* x *Sex* +  *Day.1* x *Age.2*) | |  | 0.44 ± 0.35 | | | 0.65 ± 0.25 | | 0.54 ± 0.21 | | - | | | -0.19 ± 0.08 | | -1.00 ± 0.19 | | -0.14 ± 0.09 |

**Table S4** Results of trapline-level Huggins’ p and c models for adult Rattus rattus caught in outside habitat (National dataset only), ranked by AICc. The top model set is presented with corresponding model-specific beta coefficient estimates (± SE) for covariates determining capture probability (p). The following covariates were included as additive (+) or interaction (x) effects: season, sex, age (Age3vs4), day of capture (Day. 1, capture probability varies on the first day of trapping), and habitat type (Habitat.2, capture probability varies between rice field habitat and village habitat). K = number of parameters, ΔAICc = difference in AICc value from top model. Reference values: Age3vs4 =age class 4; Day.1 = days 2-5; Habitat.2 = village; Sex = female; Season = dry. n captures = 372; n traplines = 101

|  |  |  |  | **Beta coefficients** | | | | | | | |
| --- | --- | --- | --- | --- | --- | --- | --- | --- | --- | --- | --- |
| **Model description** | **K** | **ΔAICc** | **Int.** | ***Day.1*** | ***Season*** | ***Sex*** | ***Age3vs4*** | ***Habitat.2*** | ***Sex* x *Day.1*** | ***Sex* x *Season*** | ***Age3vs4* x *Habitat.2*** |
| p(*Day.1* x *Sex* + *Season* x *Sex* + *Age3vs4* x Habitat.2) | 9 | 0 | -2.17 ± 2.12 | -0.00 ± 0.36 | 0.6 ± 0.94 | 1.23 ± 1.37 | 1.59 ± 1.33 | 0.78 ± 1.32 | -0.03 ± 0.44 | -1.47 ± 1.28 | -1.9 ± 1.64 |
| p(*Day.1* x *Sex* + *Season* x *Sex* + *Age3vs4*) | 7 | 0.37 | -1.22 ± 1.17 | 0.02 ± 0.39 | -0.14 ± 0.76 | 0.72 ± 1.08 | 0.7 ± 0.64 | - | -0.1 ± 0.48 | -0.58 ± 0.97 | - |
| p(*Day.1* x *Sex* + *Season* x *Sex* + *Age3vs4* + Habitat.2) | 8 | 0.55 | -1.1 ± 1.33 | 0.02 ± 0.39 | 0.04 ± 0.74 | 0.66 ± 1.13 | 0.8 ± 0.57 | -0.63 ± 0.57 | -0.04 ± 0.45 | -0.76 ± 1.01 | - |

**Table S5** Results of trapline-level Huggins’ p and c models for adult Rattus rattus caught in outside habitat (Antsirabe/Betafo dataset only), ranked by AICc. Top model set presented with corresponding model-specific beta coefficient estimates (± SE) for covariates determining capture probability (p). Covariates included season, sex, age (Age3vs4), day of capture (Day. 1, capture probability varies on the first day of trapping), and habitat type (Habitat.rice, capture probability varies between rice field habitat and habitat within the village and household proximity). K = number of parameters, ΔAICc = difference in AICc value from top model. The operators + and x represent additive and interaction terms. Reference values: Age3vs4 =age class 4; Day.1 = days 2-5; Rice.fields = Village and Household proximity; Sex = female; Season = dry. n captures = 1,648; n traplines = 172.

|  |  |  |  | **Beta coefficients** | | | | | | | |
| --- | --- | --- | --- | --- | --- | --- | --- | --- | --- | --- | --- |
| **Model description** | **K** | **ΔAICc** | **Int.** | ***Day.1*** | | ***Season*** | | ***Sex*** | | ***Age3vs4*** | ***Rice.fields*** |
| p(*Day.1* x *Sex* + *Season* x *Sex* + *Age3vs4* + *Rice.fields*) | 8 | 0 | -2.59 ± 1.28 | 0.32 ± 0.14 | | 1.48 ± 1.07 | | 1.72 ± 1.32 | | -0.26 ± 0.3 | 0.46 ± 0.32 |
| p(*Day.1* x *Sex* + *Season* x *Sex* + *Age3vs4*) | 7 | 0.36 | -2.62 ± 1.77 | 0.35 ± 0.15 | | 1.72 ± 1.55 | | 2.1 ± 1.82 | | -0.32 ± 0.3 | - |
| p(*Day.1* x *Sex* + *Season* x *Sex* + *Age3vs4* x *Rice.fields*) | 9 | 0.86 | -2.15 ± 1.02 | 0.3 ± 0.14 | | 1.29 ± 0.82 | | 1.51 ± 1.05 | | -0.65 ± 0.47 | 0.1 ± 0.43 |
| p(*Day.1* x *Sex* + *Season* x *Sex* + *Age3vs4* + *Sex* x *Rice.fields*) | 9 | 1.64 | -2.8 ± 1.46 | 0.34 ± 0.14 | | 1.53 ± 1.15 | | 2.08 ± 1.53 | | -0.26 ± 0.3 | 0.65 ± 0.49 |
| p(*Day.1* x *Sex* + *Season* x *Sex* + *Age3vs4* + *Season* x *Rice.fields*) | 9 | 1.97 | -2.61 ± 1.27 | 0.32 ± 0.14 | | 1.54 ± 1.1 | | 1.72 ± 1.3 | | -0.26 ± 0.3 | 0.53 ± 0.47 |
|  |  |  | ***Sex* x *Day.1*** | | ***Sex* x *Season*** | | ***Age3vs4* x  *Habitat.rice*** | | ***Sex* x  *Rice.fields*** | | ***Season* x  *Rice.fields*** |
| p(*Day.1* x *Sex* + *Season* x *Sex* + *Age3vs4* + *Rice.fields*) |  |  | -0.2 ± 0.21 | | -2.48 ± 1.21 | | - | | - | | - |
| p(*Day.1* x *Sex* + *Season* x *Sex* + *Age3vs4*) |  |  | -0.26 ± 0.21 | | -2.56 ± 1.61 | | - | | - | | - |
| p(*Day.1* x *Sex* + *Season* x *Sex* + *Age3vs4* x *Rice.fields*) |  |  | -0.2 ± 0.21 | | -2.21 ± 0.97 | | 0.63 ± 0.6 | | - | | - |
| p(*Day.1* x *Sex* + *Season* x *Sex* + *Age3vs4* + *Sex* x *Rice.fields*) |  |  | -0.24 ± 0.21 | | -2.45 ± 1.26 | | - | | -0.38 ± 0.63 | | - |
| p(*Day.1* x *Sex* + *Season* x *Sex* + *Age3vs4* + *Season* x *Rice.fields*) |  |  | -0.2 ± 0.21 | | -2.46 ± 1.19 | | - | | - | | -0.12 ± 0.6 |

**Table S6** Results of trapline-level Huggins’ p and c models for adult Rattus rattus caught in outside habitat (Moramanga dataset only), ranked by AICc. Top model set presented with corresponding model-specific beta coefficients (± SE) for covariates determining capture probability (p). Covariates included season, sex, age (Age3vs4), day of capture (Day. 1, capture probability varies on the first day of trapping), habitat type (House.prox, capture probability varies between the house proximity, forest sites, and all other habitat; or Habitat.4, capture probability varies between the house proximity, forest sites, other natural habitat, and agriculture). K = number of parameters, ΔAICc = difference in AICc value from top model. The operators + and x represent additive and interaction terms. Reference values: Age3vs4 =age class 4; Day.1 = days 2-5; Sex = female; Season = dry; House.prox = other habitat; Habitat.4 = agriculture. n captures = 3,132; n traplines = 515

|  |  |  |  | **Beta coefficients** | | | | | | | | |
| --- | --- | --- | --- | --- | --- | --- | --- | --- | --- | --- | --- | --- |
| **Model description** | **K** | **ΔAICc** | **Int.** | ***Day.1*** | ***Season*** | | ***Sex*** | ***Age3vs4*** | | ***Sex* x *Day.1*** | | ***Sex* x *Season*** |
| p(*Day.1* x *Sex* + *Season* x *Sex* + *Age3vs4* + *Day.1* x *House.prox*) | 11 | 0.00 | -1.6 ± 0.21 | -0.02 ± 0.09 | 0.67 ± 0.16 | | 0.25 ± 0.19 | 0.5 ± 0.15 | | -0.26 ± 0.11 | | -0.74 ± 0.22 |
| p(*Day.1* x *Sex* + *Season* x *Sex* + *Age3vs4* + *House.prox*) | 9 | 0.66 | -1.66 ± 0.22 | 0.03 ± 0.09 | 0.69 ± 0.17 | | 0.29 ± 0.2 | 0.5 ± 0.15 | | -0.27 ± 0.12 | | -0.77 ± 0.22 |
|  |  |  | | ***House.prox***  **(house prox)** | | ***House.prox*  (forest site)** | | | ***Day.1* x  *House.prox***  **(house prox)** | | ***Day.1* x  *House.prox*  (forest site)** | |
| p(*Day.1* x *Sex* + *Season* x *Sex* + *Age3vs4* + *Day.1* x *House.prox*) |  |  | | -0.66 ± 0.51 | | -1.16 ± 0.49 | | | -0.03 ± 0.27 | | 0.38 ± 0.18 | |
| p(*Day.1* x *Sex* + *Season* x *Sex* + *Age3vs4* + *House.prox*) |  |  | | -0.71 ± 0.39 | | -0.57 ± 0.22 | | | - | | - | |

**Table S7** Results of trapline-level Huggins’ p and c models for sub-adult Rattus rattus caught in outside habitat, grouped by dataset and ranked by AICc. Top model sets are presented with corresponding model-specific beta coefficient estimates (± SE) for the following covariates determining capture probability: sex, season, day of capture (Day. 1, capture probability varies on the first day of trapping), and habitat type (capture probability varies between rice field habitat and habitat within the village and household proximity (Habitat.rice); within the household proximity (Habitat.house); or between forest sites, other natural habitat, and all other habitat (Natural.habitat)). K = number of parameters, ΔAICc = difference in AICc value from top model. Reference values: Day.1 = days 2-5; Sex = female; Season = dry; Rice.fields = Village and Household proximity; House.prox.AB = Village and Rice field; Natural.habitat = other habitat.

|  |  |  |  | | **Beta coefficients** | |  | | |  | |  |
| --- | --- | --- | --- | --- | --- | --- | --- | --- | --- | --- | --- | --- |
| **Model description** | **K** | **ΔAICc** | **Int.** | ***Day.1*** | ***Season*** | ***Sex*** | | ***Rice.fields*** | ***House.prox.AB*** | ***Natural.habitat* (forest site)** | ***Natural.habitat* (natural)** |  |
| *Antsirabe/Betafo dataset* (n captures = 600 captures; n traplines = 172) | | | | | | | | | | | | |
| p(*Day.1* + *Sex* + *Season*) | 4 | 0 | -1.16 ± 0.57 | -0.17 ± 0.17 | -0.7 ± 0.83 | -0.05 ± 0.52 | | - | - | - | - |  |
| p(*Day.1* + *Sex* + *Season* + *Rice.fields*) | 5 | 0.37 | -0.8 ± 0.51 | -0.17 ± 0.17 | -0.59 ± 0.71 | -0.26 ± 0.54 | | -0.57 ± 0.6 | - | - | - |  |
| p(*Day.1* + *Sex* + *Season* + *House.prox.AB*) | 5 | 0.55 | -1.16 ± 0.57 | -0.17 ± 0.17 | -0.7 ± 0.86 | -0.05 ± 0.53 | | - | -0.15 ± 1.5 | - | - |  |
| *Moramanga dataset* (n captures = 1,722 captures; n traplines = 515) | | | | | | | | | | | | |
| p(*Day.1* + *Sex* + *Season*) | 4 | 0 | -2.09 ± 0.27 | -0.16 ± 0.08 | 0.64 ± 0.31 | 0.13 ± 0.28 | | - | - | - | - |  |
| p(*Day.1* + *Sex* + *Season* + *Natural.habitat*) | 6 | 2.19 | -2.44 ± 0.46 | -0.16 ± 0.08 | 0.7 ± 0.31 | 0.14 ± 0.28 | | - | - | 0.56 ± 0.49 | 0.4 ± 0.39 |  |

## References

Arnold, T. W. (2010). Uninformative Parameters and Model Selection Using Akaike’s Information Criterion. *Journal of Wildlife Management*, **74**(6), 1175–1178. <https://doi.org/10.2193/2009-367>

Burnham, K. P., & Anderson, D. R. (2002). Model Selection and Multimodel Inference: A Practical Information-Theoretic Approach (2nd ed). In Ecological Modelling (Vol. 172). https://doi.org/10.1016/j.ecolmodel.2003.11.004

Cooch, E. & White, G. (2018). Building and comparing models, in Cooch, E. & White, G. (Eds) Program MARK: A Gentle Introduction, 18th edition. Chapter 4, pp 86. Available at <http://www.phidot.org/software/mark/docs/book/>

Funk, C., Peterson, P., Landsfeld, M., *et al.* (2015). The climate hazards infrared precipitation with stations—a new environmental record for monitoring extremes. Scientific Data, vol. 2, Article ID 150066.

Leroux, S. J. (2019). On the prevalence of uninformative parameters in statistical models applying model selection in applied ecology. *PLoS ONE*, **14**(2), 1–12.

Lukacs, P. (2018). Closed population capture-recapture models, in Cooch, E. & White, G. (Eds) Program MARK: A Gentle Introduction, 18th edition. Chapter 14.6, pp 15. Available at <http://www.phidot.org/software/mark/docs/book/>

Richards, S. A., Whittingham, M. J., & Stephens, P. A. (2011). Model selection and model averaging in behavioural ecology: The utility of the IT-AIC framework. Behavioral Ecology and Sociobiology, 65(1), 77–89. <https://doi.org/10.1007/s00265-010-1035-8>

White, G.C. & Burnham, K.P. (1999) Program MARK: survival estimation from populations of marked animals. *Bird Study*, **46**, S120–S139.
