# Supplementary material for: Reproductive ecology of the black rat (Rattus rattus) in Madagascar: the influence of density‐dependent and ‐independent effects
Source: Integr Zool. 2023 Jul 11;19(1):66–86. doi: 10.1111/1749-4877.12750 (PMC10952345; doi:10.1111/1749-4877.12750)
Supplement: Supplementary file 2 — Figure S1 Flow diagram illustrating the three‐stage process followed during model selection. Figure S2 Capture rate of female Rattus rattus (≥45 g) grouped by (a) dataset and bioclimate and (b) month and habitat type. Figure S3 (a–d) QQ‐plot (left) and plot of standardized residuals versus model predictions (right) simulated from the fitted models of Rattus rattus reproductive rates outside of houses (Table S17). Red lines indicate quantile deviations detected. Figure S4 (a–d) QQ‐plot (left) and plot of standardized residuals vs model predictions (right) simulated from the fitted models of Rattus rattus reproductive rates inside houses (Table S18). Red lines indicate quantile deviations detected. Figure S5 (a–d) QQ‐plot (left) and plot of standardized residuals vs model predictions (right) simulated from the fitted models of Rattus rattus reproductive rates outside of houses (Table 3, main text). Red lines indicate quantile deviations detected. Figure S6 (a–c) QQ‐plot (left) and plot of standardized residuals vs model predictions (right) simulated from the fitted models of Rattus rattus reproductive rates inside houses (Table 4, main text). Red lines indicate quantile deviations detected. [file INZ2-19-66-s001.pdf]

Stage 0

Prior to model selection, we identified which variables (and interactions) to include in a global model based on a set of a priori hypotheses (H_A1-8):

H_A1: Spatial and temporal variation in climate influences primary productivity and thus resource availability, impacting reproductive rates (*Bioclimate* + *Season)*

H_A2: Outside of houses, resource availability is influenced by proximity to houses and agricultural habitat (*Habitat*)

H_A3-H_A5: The influence of rainfall on primary productivity influences reproductive rates (*Rainfall*); the effect of rainfall varies spatially (*Rainfall* x *Bioclimate*), seasonally (*Rainfall* x *Season*), and between different habitats (*Rainfall* x *Habitat*)

H_A6-H_A8: Density dependent effects influence reproductive activity (*Density*); these effects vary seasonally (*Density* x *Season*), between different habitats (*Density x Habitat*), and between individuals of different ages (*Density* x *Head-body length (HBL)*)

(Global Model 1) Reproduction outside of houses ~ (*Rainfall* x *Season*) + (*Rainfall* x *Bioclimate*) + (*Rainfall* x *Habitat*) + (*Density* x *Season*) + (*Density x Habitat*) + (*Density* x *HBL)*

(Global Model 2) Reproduction inside houses ~ (*Rainfall* x *Season*) + (*Rainfall* x *Bioclimate*) + (*Density* x *Season*) + (*Density* x *HBL*)

Diagram Key

- Stage 0 - generating a priori hypotheses and construction of global models
- Stage 1 - variable selection
- Stage 2 - assessing simplifications of each global model
- Stage 3 - identifying final models

Stage 1

In Stage 1, we tested different characterisations of rainfall, bioclimate, density and habitat variables:

(a) For **outside and in-house** models, we ran the following models:

Bioclimate: *Bio.2* *Bio.3* *Bio.4* *Bio.5*

Rainfall: *Lag.0* *Acc.lag.0* *Lag.1* *Acc.lag.1* *Lag.2* *Acc.lag.2* *Lag.3* *Acc.lag.3*

Density: *Site.density* *Household.site.level* *Trapline.density* *Household.capture.rate*

For **outside models only**, we also tested:

Habitat: *Habitat* *Household_proximity* *Within_village.vs.Outside_village*

+/x *Season*

(b) Characterisations of rainfall and density appearing in models with ΔAICc ≤2 were further tested in the following models:

Rainfall x Bioclimate Density x HBL

(c) Models containing **different characterisations** of the same variable were compared using AICc values. Characterisations which generated models with ΔAICc ≤2 were retained and used to construct a set of global models (using the pre-defined structure outlined in Stage 0).

Box 1

For example, to identify which rainfall and bioclimate characterisations to include in models of female maturity outside of houses:

(1a) **16 models** are run containing the following explanatory variables, and compared using (ΔAICc):

Lag.0 + *Season* (9.9)	*Acc.lag.0* + *Season* (12.3)
Lag.0 x *Season* (6.2)	*Acc.lag.0* x *Season* (4.0)
Lag.1 + *Season* (9.4)	*Acc.lag.1* + *Season* (7.5)
Lag.1 x *Season* (5.5)	*Acc.lag.1* x *Season* (2.4)
Lag.2 + *Season* (9.9)	*Acc.lag.2* + *Season* (12.8)
Lag.2 x *Season* (6.4)	***Acc.lag.2* x *Season* (0.0)**
Lag.3 + *Season* (12.5)	*Acc.lag.3* + *Season* (6.8)
Lag.3 x *Season* (4.7)	*Acc.lag.3* x *Season* (3.4)

(2b) ***Acc.lag.2*** generated the only model with ΔAICc ≤2 and is further tested in the following **4 models**:

***Acc.lag.2* x *Bio.2* (0.0)**	***Acc.lag.2* x *Bio.4* (0.8)**
Acc.lag.2 x *Bio.3* (3.8)	*Acc.lag.2* x *Bio.5* (4.6)

(2c) The variables ***Acc.lag.2***, ***Bio.2*** and ***Bio.4*** are retained and used to construct a set of global models (n=2):

GM1: Maturity ~ (***Acc.lag.2*** x *Season*) + (***Acc.lag.2*** x ***Bio.2***) + (***Acc.lag.2*** x *Habitat*) + (*Density* x *Season*) + (*Density* x *Habitat*) + (*Density* x *HBL*)

and

GM2: Maturity ~ (***Acc.lag.2*** x *Season*) + (***Acc.lag.2*** x ***Bio.4***) + (***Acc.lag.2*** x *Habitat*) + (*Density* x *Season*) + (*Density* x *Habitat*) + (*Density* x *HBL*)

Stage 2

In Stage 2, we assessed simplifications of each global model using the "dredge" function in R's MuMIn package.

(a) The dredge function is used to assess every combination of variables included within each of the global models, generating sets of candidate models.

If more than one characterisation of the same variable are retained after Stage 1 of model selection, then multiple global models are run.

(b) Candidate models are **grouped by response variable** and ranked based on AICc.

Candidate model sets may therefore include models which contain different characterisations of the same variable.

(c) Candidate model sets are subset to include only models with ΔAICc ≤2. This subset of models is referred to as **"top model set"**:

Box 2

When modelling female maturity outside of houses, **two characterisations of bioclimate** were retained after Stage 1 of model selection (*Bio.2* and *Bio.4*) and used to construct two global models (**GM1** and **GM2**).

(2a) Models GM1 and GM2 are 'dredged', generating two sets of candidate models. One set incldues the bioclimate variable ***Bio.2*** and the second set includes the biocliamte variable ***Bio.4.***

(2b) The two sets of candidate models are combined and ranked by AICc:

(The top 5 candidate models are presented)

m1: (*Acc.lag.2* x *Season*) + ***Bio.4*** + (*Acc.lag.2* x *Habitat*) + *Density* + *HBL* (AICc= 2496.6)

m2: (*Acc.lag.2* x *Season*) + (*Acc.lag.2* x ***Bio.2***) + (*Acc.lag.2* x *Habitat*) + *Density* + *HBL* (2497.0)

m3: (*Acc.lag.2* x *Season*) + (*Acc.lag.2* x *Habitat*) + *Density* + *HBL* (2497.0)

m4: (*Acc.lag.2* x *Season*) + ***Bio.4*** + (*Acc.lag.2* x *Habitat*) + (*Habitat* x *Density*) + *HBL* (2497.8)

m5: (*Acc.lag.2* x *Season*) + *Density* + *HBL* (2500.5)

...

(2c) Models m1, m2, m3 and m4 have ΔAICc ≤2 and are retained in a "top model set".

Stage 3

In Stage 3, we identified which model(s) from the top model set were most informative

(a) Models containing uninformative parameters are removed from the top model set using the following selection criteria.

I. Models containing alternative characterisations of a variable included in a model with a lower AICc are removed;

II. Models that differ from other models with a lower AICc only by the inclusion of a single parameter for which the 95% confidence intervals overlapped zero are removed

III. Models that are nested within the model with the lowest AICc are removed

(b) Models remaining in the top model set includes the final models considered to be most informative for interpretation.

Box 3

(3a) Following the selection criteria outlined above, three models are removed from our top model set:

I. Model m2 is removed from the top model set as a model with a lower AICc (m1) contains an alternative version of bioclimate:

m1: (*Acc.lag.2* x *Season*) + ***Bio.4*** + (*Acc.lag.2* x *Habitat*) + *Density* + *HBL* (AICc= 2496.6)

m2: (*Acc.lag.2* x *Season*) + (*Acc.lag.2* x ***Bio.2***) + (*Acc.lag.2* x *Habitat*) + *Density* + *HBL* (2497.0)

II. Model m4 differs from m1 by the inclusion of a single parameter: (*Habitat* x *Density*)

The 95% CIs for this interaction overlap 0 **(-0.02 - 0.06)** and so m4 is removed from the top model set.

III. Model **m3 is nested in m1** - the model with the lowest AICc - and is removed from the top model set

(3b) The final model remaining is:

m1: (*Acc.lag.2* x *Season*) + ***Bio.4*** + (*Acc.lag.2* x *Habitat*) + *Density* + *HBL*

Figure S1. *Flow diagram illustrating the three-stage process followed during model selection. Illustrative examples are provided in grey boxes (Box 1-3). In Box 2, a subset of the candidate models (generated after dredging the two global models run for female maturity outside of houses) are presented (m1-m5). `Additive and interaction effects are represented by the following notations: '+'* and *'x'.*
