# Supplementary material for: Reproductive ecology of the black rat (Rattus rattus) in Madagascar: the influence of density‐dependent and ‐independent effects
Source: Integr Zool. 2023 Jul 11;19(1):66–86. doi: 10.1111/1749-4877.12750 (PMC10952345; doi:10.1111/1749-4877.12750)
Supplement: Supplementary file 3 — Table S9–Table S18 Results of GLMM analysis of variables predicting reproductive rates of female Rattus rattus inside houses. [file INZ2-19-66-s005.docx]

**Supplementary Materials – Capture summary**


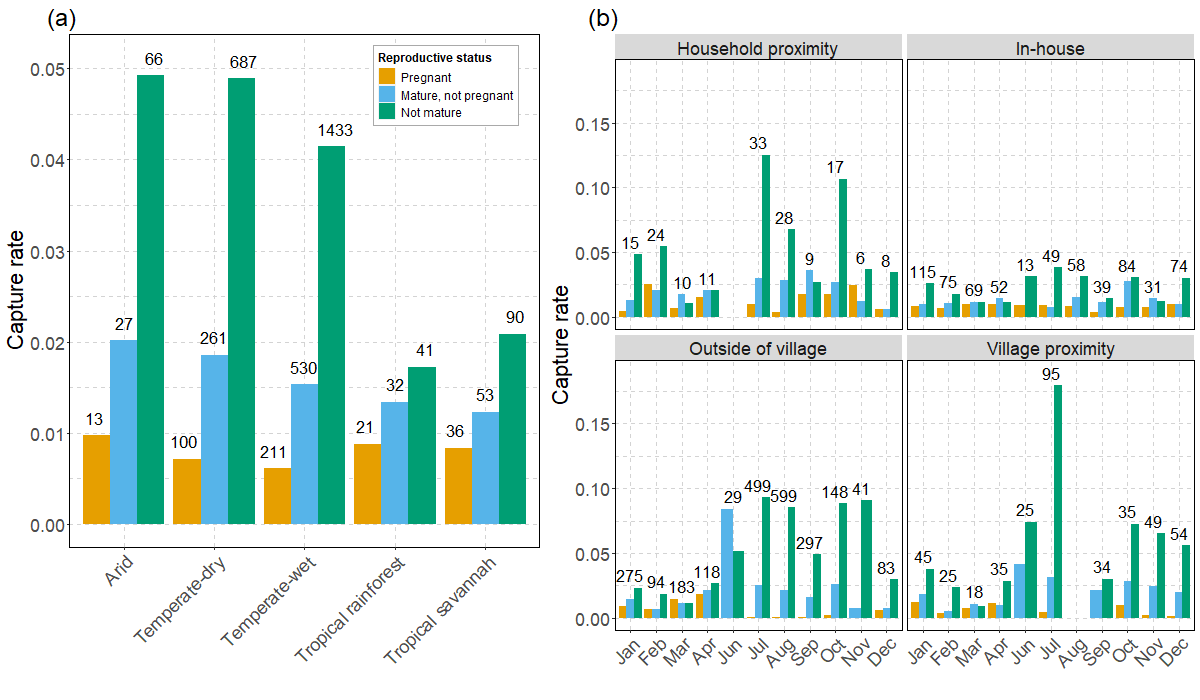


**Figure S2** Capture rate of female R. rattus (≥45 g) grouped by **a** dataset and bioclimate and **b** month and habitat type. Capture rate is calculated as the number of captures divided by the number of traps containing rodents or not sprung plus half the number of traps which were sprung or had bait removed but which had not caught a rodent (Theuerkauf et al., 2011). Sample sizes are shown above bars and represent **a** total number of captures grouped by reproductive status or **b** sum of all captures combined (i.e. all mature and non-mature individuals >45 g).
