# Supplementary material for: Reproductive ecology of the black rat (Rattus rattus) in Madagascar: the influence of density‐dependent and ‐independent effects
Source: Integr Zool. 2023 Jul 11;19(1):66–86. doi: 10.1111/1749-4877.12750 (PMC10952345; doi:10.1111/1749-4877.12750)

**Supplementary Materials –** **Preliminary analysis model selection results and top model sets**

**Table S9** Evaluation of different habitat characterisations for GLMM analysis of female Rattus rattus reproductive rates outside of houses. ‘Habitat’ characterises habitat type as either: household proximity, village proximity, or outside of the village. In ‘Household.proximity’, habitat in the village proximity and outside of the village are grouped. Models included an interaction between habitat and season, an additive effect of head-body length, and a random effect of site and mission nested within site. Models in the same row were compared using Akaike’s Information Criterion corrected for small sample size (AICc). All models within 2 of the lowest AICc value (bold) were included in subsequent global models.

|  | Interaction | |
| --- | --- | --- |
|  | *Habitat* | *Household.proximity* |
| Female maturity | **2683.0** | 2691.0 |
| Gestation rate | 874.1 | **871.7** |
| Litter size | 1031.4 | **1028.6** |

**Table S10** Evaluation of different bioclimate characterisations for GLMM analysis of female Rattus rattus reproductive rates. Each characterisation was assessed as an additive effect and in interaction with season. Models also included an additive effect of head-body length and a random effect of site and mission nested within site. Models in the same row were compared using corrected Akaike’s Information Criterion (AICc). All models within 2 of the lowest AICc value were included in subsequent global models except if a model with an interaction was within 2 AICc of the equivalent additive model, in which case only the interaction effect was included in the global model (bold).

|  | Additive | | | | Interaction | | | |
| --- | --- | --- | --- | --- | --- | --- | --- | --- |
|  | *Bio.2* | *Bio.3* | *Bio.4* | *Bio.5* | *Bio.2* | *Bio.3* | *Bio.4* | *Bio.5* |
| *Outside of houses* | | | | | | | | |
| Female maturity | **2688.4** | 2690.4 | **2688.8** | 2690.7 | **2687.9** | **2687.5** | **2687.9** | **2687.0** |
| Gestation rate | 883.4 | **883.2** | 883.9 | 883.7 | **881.2** | **882.4** | 883.5 | 884.5 |
| Litter size | **1027.0** | 1029.2 | 1029.9 | 1032.1 | 1031.3 | 1034.4 | 1040.9 | 1044.2 |
| *Inside houses* | | | | | | | | |
| Female maturity | **733.5** | 735.6 | **733.7** | 735.8 | 735.5 | **733.1** | 739.9 | 737.2 |
| Gestation rate | 435.7 | 436.6 | 438.5 | 439.6 | **427.9** | 434.0 | 439.5 | 445.8 |
| Litter size | **545.9** | **545.3** | 549.8 | 549.1 | 548.3 | 551.7 | 558.7 | 562.7 |

**Table S11** AICc values and standardized regression coefficients from preliminary GLMM analysis of bioclimate and habitat variables predicting maturity in female Rattus rattus outside of houses. Models in the top model set are presented (m1-6) (i.e., models with an AICc within 2 of the lowest AICc but excluding those which include only one of either Season.sin or Season.cos). Models m1 and m3 were selected as the final models. ^ⴕ^Confidence intervals overlap zero. Reference level: Bioclimate = Arid. Habitat = Outside of village.

|  | **m1** | **m2** | **m3** | **m4** | **m5** | **m6** |
| --- | --- | --- | --- | --- | --- | --- |
| AICc | 2683.0 | 2684.3 | 2683.0 | 2683.5 | 2684.4 | 2684.5 |
| Log likelihood | -1329.4 | -1329.1 | -1326.4 | -1320.6 | -1326.1 | .1318.1 |
| **β coefficients** |  |  |  |  |  |  |
| Intercept | -0.78 | -0.44^ⴕ^ | -1.93 | -1.02^ⴕ^ | -1.97 | -1.03^ⴕ^ |
| *Season.sin* | 1.22 | 1.23 | 1.21 | 1.78^ⴕ^ | 1.23 | 1.69^ⴕ^ |
| *Season.cos* | -0.1^ⴕ^ | -0.13^ⴕ^ | -0.15^ⴕ^ | -3.12^ⴕ^ | -0.15^ⴕ^ | -3.29^ⴕ^ |
| *Habitat* : Household proximity | 0.07^ⴕ^ | 0.05^ⴕ^ | 0.03^ⴕ^ | 0.02^ⴕ^ | 0.04^ⴕ^ | 0.04^ⴕ^ |
| *Habitat* : Village proximity | 0.21^ⴕ^ | 0.19^ⴕ^ | 0.23^ⴕ^ | 0.21^ⴕ^ | 0.28^ⴕ^ | 0.25^ⴕ^ |
| *Length.cont* | 1.5 | 1.5 | 1.51 | 1.5 | 1.5 | 1.5 |
| Season.cos x *Habitat* : |  |  |  |  |  |  |
| Household proximity | 0.19^ⴕ^ | 0.17^ⴕ^ | 0.17^ⴕ^ | 0.18^ⴕ^ | 0.17^ⴕ^ | 0.2^ⴕ^ |
| Village proximity | 0.1^ⴕ^ | 0.09^ⴕ^ | 0.11^ⴕ^ | 0.13^ⴕ^ | 0.11^ⴕ^ | 0.3^ⴕ^ |
| *Season.sin* x *Habitat* : |  |  |  |  |  |  |
| Household proximity | -0.69 | -0.7 | -0.71 | -0.72 | -0.71 | -0.72 |
| Village proximity | -1.03 | -1.02 | -1.08 | -1.06 | -1.09 | -0.95 |
| Bioclimate |  |  |  |  |  |  |
| Temperate (all) | - | -0.37^ⴕ^ | 1.11^ⴕ^ | 0.19^ⴕ^ | - | - |
| Temperate-dry | - | - | - | - | 1.0^ⴕ^ | 0.12^ⴕ^ |
| Temperate-wet | - | - | - | - | 1.24^ⴕ^ | 0.3^ⴕ^ |
| Tropical (rainforest) | - | - | 2.37 | 3.2^ⴕ^ | 2.38 | 3.18^ⴕ^ |
| Tropical (savannah) | - | - | 1.78^ⴕ^ | 2.95^ⴕ^ | 1.8^ⴕ^ | 2.96^ⴕ^ |
| *Season.cos* x Bioclimate |  |  |  |  |  |  |
| Temperate (all) | - | - | - | 3.04^ⴕ^ | - | - |
| Temperate-dry | - | - | - | - | - | 2.79^ⴕ^ |
| Temperate-wet | - | - | - | - | - | 3.45^ⴕ^ |
| Tropical (rainforest) | - | - | - | 0.48^ⴕ^ | - | 0.62^ⴕ^ |
| Tropical (savannah) | - | - | - | -0.38^ⴕ^ | - | -0.28^ⴕ^ |
| *Season.sin* x Bioclimate |  |  |  |  |  |  |
| Temperate (all) | - | - | - | -0.56^ⴕ^ | - | - |
| Temperate-dry | - | - | - | - | - | -0.78^ⴕ^ |
| Temperate-wet | - | - | - |  | - | -0.41^ⴕ^ |
| Tropical (rainforest) | - | - | - | -2.37^ⴕ^ | - | -2.31^ⴕ^ |
| Tropical (savannah) | - | - | - | -2.15^ⴕ^ | - | -2.07^ⴕ^ |

**Table S12** AICc values and standardized regression coefficients from preliminary GLMM analysis of bioclimate and habitat variables predicting gestation rate of sexually mature Rattus rattus outside of houses. Top model set is presented (m1-6) (i.e., models with an AICc within 2 of the lowest AICc but excluding those which include only one of either Season.sin or Season.cos). Model m3 was selected as the final model. ^ⴕ^Confidence intervals overlap zero. Reference level: Bioclimate = Arid. Household.proximity reference level = Village proximity and outside of village.

|  | **m1** | **m2** | **m3** | **m4** | **m5** | **m6** |
| --- | --- | --- | --- | --- | --- | --- |
| AICc | 866.2 | 867.7 | 865.7 | 866.5 | 866.9 | 867.2 |
| Log likelihood | -421.98 | -421.68 | -415.53 | -414.88 | -422.33 | -421.413 |
| **β coefficients** |  |  |  |  |  |  |
| Intercept | -0.82^ⴕ^ | -0.82^ⴕ^ | -0.27^ⴕ^ | -0.23^ⴕ^ | -0.^81ⴕ^ | 0.82^ⴕ^ |
| *Season.sin* | 0.58^ⴕ^ | 0.56^ⴕ^ | -3.84^ⴕ^ | -4.02^ⴕ^ | 1.6 | 1.61 |
| *Season.cos* | 0.39^ⴕ^ | 0.33^ⴕ^ | -10.47^ⴕ^ | -10.92^ⴕ^ | 1.14 | 1.11 |
| *Household.proximity* : Household proximity | 0.92 | 0.93 | 1.12 | 1.14 | 0.93 | 0.92 |
| *Length.cont* | - | -0.07^ⴕ^ | - | -0.11^ⴕ^ | - | - |
| *Season.cos* x *Household.proximity* | - | - | -0.26^ⴕ^ | -0.29^ⴕ^ | -0.29^ⴕ^ | -0.22^ⴕ^ |
| *Season.sin* x *Household.proximity* | - | - | -1.79 | -1.78 | -1.74 | -1.73 |
| Bioclimate |  |  |  |  |  |  |
| Temperate (all) | -1.03^ⴕ^ | -1.03 | -1.61^ⴕ^ | -1.66^ⴕ^ | -1^ⴕ^ | - |
| Temperate-dry | - | - | - | - | - | -1.19^ⴕ^ |
| Temperate-wet | - | - | - | - | - | -0.86^ⴕ^ |
| Tropical (rainforest) | - | - | -0.41^ⴕ^ | -0.42^ⴕ^ | 0.52^ⴕ^ | 0.54^ⴕ^ |
| Tropical (savannah) | - | - | -1.45^ⴕ^ | -1.54^ⴕ^ | -1.36^ⴕ^ | -1.35^ⴕ^ |
| *Season.cos* x Bioclimate |  |  |  |  |  |  |
| Temperate (all) | - | - | 11.56^ⴕ^ | 12.03^ⴕ^ | - | - |
| Temperate-dry | - | - | - | - | - | - |
| Temperate-wet | - | - | - | - | - | - |
| Tropical (rainforest) | - | - | 13.31^ⴕ^ | 13.73^ⴕ^ | - | - |
| Tropical (savannah) | - | - | 10.31^ⴕ^ | 10.75^ⴕ^ | - | - |
| *Season.sin* x Bioclimate |  |  |  |  |  |  |
| Temperate (all) | - | - | 5.62^ⴕ^ | 5.8^ⴕ^ | - | - |
| Temperate-dry | - | - | - | - | - | - |
| Temperate-wet | - | - | - |  | - | - |
| Tropical (rainforest) | - | - | 4.04^ⴕ^ | 4.16 ^ⴕ^ | - | - |
| Tropical (savannah) | - | - | 5.46^ⴕ^ | 5.64^ⴕ^ | - | - |

**Table S13** AICc values and standardized regression coefficients from preliminary GLMM analysis of bioclimate and habitat variables predicting litter size of pregnant Rattus rattus outside of houses. Models within the top model set are presented (m1-2) (i.e., models with an AICc within 2 of the lowest AICc but excluding those which include only one of either Season.sin or Season.cos). Model m1 was selected as the final model. ^ⴕ^Confidence intervals overlap zero. Household.proximity reference level = Village proximity and outside of village.

|  | **m1** | **m2** |
| --- | --- | --- |
| AICc | 1024.9 | 1026.0 |
| Log likelihood | -505.2 | -504.7 |
| **β coefficients** |  |  |
| Intercept | 1.67 | 1.66 |
| *Season.sin* | 0.21 | 0.22 |
| *Season.cos* | 0.0^ⴕ^ | 0.01^ⴕ^ |
| *Length.cont* | 0.08 | 0.08 |
| *Household.proximity : House proximity* | - | 0.07^ⴕ^ |

**Table S14** AICc values and standardized regression coefficients from preliminary GLMM analysis of bioclimate and habitat variables predicting maturity in female Rattus rattus inside houses. Models within the top model set are presented (m1-4) (i.e., models with an AICc within 2 of the lowest AICc but excluding those which include only one of either Season.sin or Season.cos). Models m1 and m2 were selected as the final models. ^ⴕ^Confidence intervals overlap zero. Bioclimate reference level = Arid and Tropical.

|  | **m1** | **m2** | **m3** | **m4** |
| --- | --- | --- | --- | --- |
| AICc | 731.6 | 733.1 | 733. 4 | 733.5 |
| Log likelihood | -359.7 | -354.3 | -362.6 | -359.7 |
| **β coefficients** |  |  |  |  |
| Intercept | -0.1^ⴕ^ | 0.36^ⴕ^ | -0.07^ⴕ^ | -0.01^ⴕ^ |
| *Season.sin* | 0.49 | 0.2^ⴕ^ | - | 0.49 |
| *Season.cos* | 0.05^ⴕ^ | -0.75^ⴕ^ | - | 0.03^ⴕ^ |
| *Length.cont* | 1.36 | 1.35 | 1.34 | 1.36 |
| Bioclimate |  |  |  |  |
| Temperate (all) | - | - | - | -0.13^ⴕ^ |
| Temperate-dry | - | -0.47^ⴕ^ | - | - |
| Temperate-wet | - | -0.37^ⴕ^ | - | - |
| *Season.cos* x Bioclimate |  |  |  |  |
| Temperate-dry | - | 0.75^ⴕ^ | - | - |
| Temperate-wet | - | 0.29^ⴕ^ | - | - |
| *Season.sin* x Bioclimate |  |  |  |  |
| Temperate-dry | - | -0.44^ⴕ^ | - | - |
| Temperate-wet | - | 1.18 | - | - |

**Table S15** AICc values and standardized regression coefficients from preliminary GLMM analysis of bioclimate and habitat variables predicting gestation rate of sexually mature female Rattus rattus inside houses. Model m1 represents the top model set (i.e., models with an AICc within 2 of the lowest AICc but excluding those which include only one of either Season.sin or Season.cos) and was selected as the final model. ^ⴕ^Confidence intervals overlap zero. Bioclimate reference level = Arid and Tropical.

|  | **m1** |
| --- | --- |
| AICc | 427.2 |
| Log likelihood | -204.3 |
| **β coefficients** |  |
| Intercept | -1.04 |
| *Season.sin* | 0.41^ⴕ^ |
| *Season.cos* | 1.24 |
| *Length.cont* | -0.28 |
| Bioclimate |  |
| Temperate (all) | 0.66 |
| *Season.cos* x Bioclimate |  |
| Temperate (all) | -1.72 |
| *Season.sin* x Bioclimate |  |
| Temperate (all) | 0.31^ⴕ^ |

**Table S16** AICc values and standardized regression coefficients from preliminary GLMM analysis of bioclimate and habitat variables predicting litter size of pregnant Rattus rattus inside houses. Models within the top model set are presented (m1-6) (i.e., models with an AICc within 2 of the lowest AICc but excluding those which include only one of either Season.sin or Season.cos). Model m1 was selected as the final model. ^ⴕ^Confidence intervals overlap zero. Bioclimate reference level = Arid and Tropical.

|  | **m1** | **m2** | **m3** | **m4** | **m5** | **m6** |
| --- | --- | --- | --- | --- | --- | --- |
| AICc | 545.0 | 545.3 | 547.0 | 545.1 | 545.3 | 546.8 |
| Log likelihood | -263.9 | -266.3 | -266.0 | -262.8 | -265.2 | -264.8 |
| **β coefficients** |  |  |  |  |  |  |
| Intercept | 1.67 | 1.68 | 1.76 | 1.66 | 1.68 | 1.63 |
| *Season.sin* | 0.09 | - | 0.09 | 0.09 | - | 0.1 |
| *Season.cos* | -0.03^ⴕ^ | - | -0.05^ⴕ^ | -0.01^ⴕ^ | - | -0.01^ⴕ^ |
| *Length.cont* | 0.06^ⴕ^ | 0.07 | 0.07 | 0.06^ⴕ^ | 0.07 | - |
| Bioclimate |  |  |  |  |  |  |
| Temperate (all) | 0.13 | 0.14 | - | - | - | - |
| Temperate-dry | - | - | - | 0.07^ⴕ^ | 0.08^ⴕ^ | 0.1^ⴕ^ |
| Temperate-wet | - | - | - | 0.18 | 0.18 | 0.22 |

**Table S17** Results of GLMM analysis of variables predicting reproductive rates of female Rattus rattus outside of houses. Standardized regression coefficients (β) and standard error (SE) are presented. Model parameters were included as additive effects or interactions (indicated by ‘x’). For models of female maturity, models 1 (m1) and 2 (m2) were considered competitive and so the results of both are presented. ^ⴕ^ Habitat reference level: Habitat = Outside of village; Household.proximity = Village proximity and Outside of village. ^‡^Bioclimate reference level: Bio.4 = Arid; Bio.2 = Arid and Tropical.

|  |  | **Female maturity** | | | | **Gestation rate** | | **Litter size** | |
| --- | --- | --- | --- | --- | --- | --- | --- | --- | --- |
|  |  | **m1** | | **m2** | |  |  |  |  |
| **Variable** | **Characterisation (level)** | **β** | **SE** | **β** | **SE** | **β** | **SE** | **β** | **SE** |
| (Intercept) |  | -0.78 | 0.14 | -1.93 | 0.79 | -0.82 | 0.47 | 1.67 | 0.04 |
| Habitat ^ⴕ^ | *Habitat* (Household proximity) | 0.07 | 0.22 | 0.03 | 0.23 | - | - | - | - |
|  | *Habitat* (Village proximity) | 0.21 | 0.19 | 0.23 | 0.19 | - | - | - | - |
|  | *Household.proximity* (Household proximity) | - | - | - | - | 0.92 | 0.33 | - | - |
| Season | *Season.sin* | 1.22 | 0.21 | 1.21 | 0.2 | 0.58 | 0.44 | 0.21 | 0.04 |
|  | *Season.cos* | -0.11 | 0.21 | -0.15 | 0.21 | 0.39 | 0.91 | 0.00 | 0.05 |
| Head-body length | *Length.cont* | 1.5 | 0.07 | 1.51 | 0.07 | - | - | 0.08 | 0.02 |
| Season x Habitat | *Season.sin* x *Habitat* (Household prox.) | -0.69 | 0.35 | -0.71 | 0.35 | - | - | - | - |
|  | *Season.sin* x *Habitat* (Village prox.) | -1.03 | 0.29 | -1.08 | 0.29 | - | - | - | - |
|  | *Season.sin* x *Household.proximity* (Household prox.) | - | - | - | - | -1.58 | 0.44 | - | - |
|  | *Season.cos* x *Habitat* (Household prox.) | 0.19 | 0.33 | 0.17 | 0.33 | - | - | - | - |
|  | *Season.cos* x *Habitat* (Village prox.) | 0.1 | 0.26 | 0.11 | 0.26 | - | - | - | - |
|  | *Season.cos* x *Household.proximity* (Household prox.) | - | - | - | - | -0.5 | 0.51 | - | - |
| Bioclimate^‡^ | *Bio.4* (Temperate) | - | - | 1.11 | 0.79 | - | - | - | - |
|  | *Bio.4* (Tropical rainforest) | - | - | 2.37 | 1.08 | - | - | - | - |
|  | *Bio.4* (Tropical savannah) | - | - | 1.78 | 0.97 | - | - | - | - |
|  | *Bio.2* (Temperate) | - | - | - | - | -1.03 | 0.49 | - | - |
| Season x Bioclimate | *Season.sin* x *Bio.2* (Temperate) | - | - | - | - | 1.15 | 0.47 | - | - |
|  | *Season.cos* x *Bio.2* (Temperate) | - | - | - | - | 0.73 | 0.92 | - | - |
| **Random effect** |  | **Variance (SD)** | | | | | |  |  |
| Site_visit : Site |  | 1.38 (1.17) | | 1.28 (1.13) | | <0.001 (<0.001) | | <0.001 (0.01) | |
| Site |  | <0.001 (<0.001) | | <0.001 (<0.001) | | 0.21 (0.45) | | 0.01 (0.1) | |
| **AICc (ΔAICc)** |  | 2682.9 (0.1) | | 2682.8 (0.0) | | 866.2 (0.0) | | 1024.9 (0.0) | |

**Table S18** Results of GLMM analysis of variables predicting reproductive rates of female Rattus rattus inside houses. Standardized regression coefficients (β) and standard error (SE) are presented. Model parameters were included as additive effects or interactions (indicated by ‘x’). For models of female maturity, models 1 (m1) and 2 (m2) were considered competitive and so the results of both are presented. ^ⴕ^ Bioclimate reference level: Bio.3 = Arid and Tropical; Bio.2 = Arid and Tropical.

|  |  | **Female maturity** | | | | **Gestation rate** | | **Litter size** | | |
| --- | --- | --- | --- | --- | --- | --- | --- | --- | --- | --- |
|  |  | **m1** | | **m2** | |  |  |  |  |  |
| **Variable** | **Characterisation (level)** | **β** | **SE** | **β** | **SE** | **β** | **SE** | **β** | **SE** |  |
| (Intercept) |  | -0.1 | 0.17 | 0.36 | 0.39 | -1.04 | 0.27 | 1.67 | 0.05 |  |
| Season | *Season.sin* | 0.49 | 0.22 | 0.2 | 0.36 | 0.41 | 0.24 | 0.09 | 0.04 |  |
|  | *Season.cos* | 0.05 | 0.22 | -0.75 | 0.6 | 1.24 | 0.44 | -0.03 | 0.05 |  |
| Head-body length | *Length.cont* | 1.36 | 0.13 | 1.35 | 0.13 | -0.29 | 0.13 | 0.06 | 0.03 |  |
| Bioclimate^ⴕ^ | *Bio.3* (Temperate-dry) | - | - | -0.47 | 0.48 | - | - | - | - |  |
|  | *Bio.3* (Temperate-wet) | - | - | -0.37 | 0.49 | - | - | - | - |  |
|  | *Bio.2* (Temperate) | - | - | - | - | 0.66 | 0.31 | 0.13 | 0.06 |  |
| Season x Bioclimate | *Season.sin* x *Bio.3* (Temperate-dry) | - | - | -0.44 | 0.52 | - | - | - | - |  |
|  | *Season.sin* x *Bio.3*(Temperate-wet) | - | - | 1.18 | 0.54 | - | - | - | - |  |
|  | *Season.sin* x *Bio.2*(Temperate) | - | - | - | - | 0.31 | 0.34 | - | - |  |
|  | *Season.cos* x *Bio.3* (Temperate-dry) | - | - | 0.75 | 0.67 | - | - | - | - |  |
|  | *Season.cos* x *Bio.3* (Temperate-wet) | - | - | 0.29 | 0.72 | - | - | - | - |  |
|  | *Season.cos* x *Bio.2* (Temperate) | - | - | - | - | -1.72 | 0.5 | - | - |  |
| **Random effect** |  | **Variance (SD)** | | | | | | | | |
| Site_mission : Site |  | 0.33 (0.57) | | <0.001 (<0.001) | | <0.001 (<0.001) | | <0.001 (<0.001) | | |
| Site |  | 0.61 (0.782) | | 0.81 (0.9) | | <0.001 (<0.001) | | <0.001 (<0.001) | | |
| **AICc (ΔAICc)** |  | 731.6 (0.0) | | 733.1 (1.5) | | 427.2 (0.0) | | 545.0 (0.0) | | |

**Figure S3 (a-d).** QQ-plot (left) and plot of standardized residuals vs model predictions (right) simulated from the fitted models of Rattus rattus reproductive rates outside of houses (Table S17). Red lines indicate quantile deviations detected.

(b) Female maturity (m2)


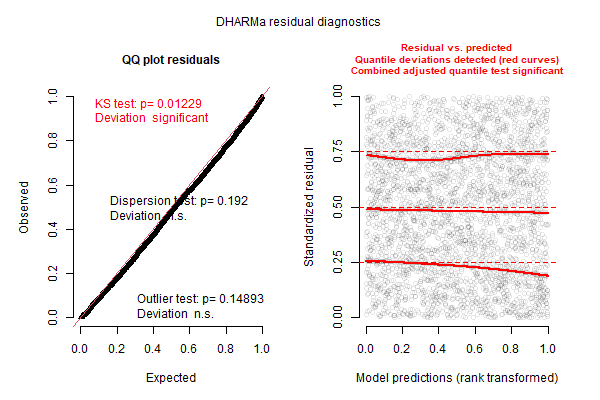


(a) Female maturity (m1)


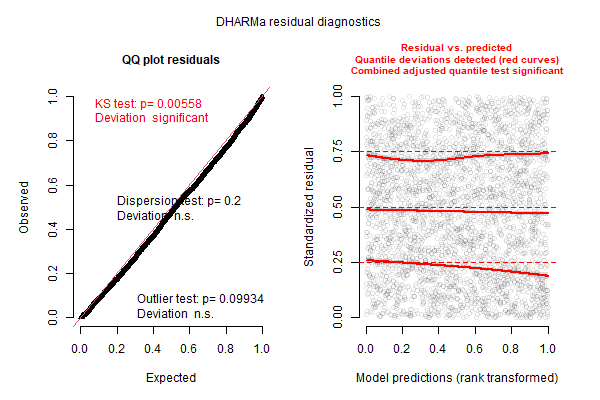


(c) Gestation rate


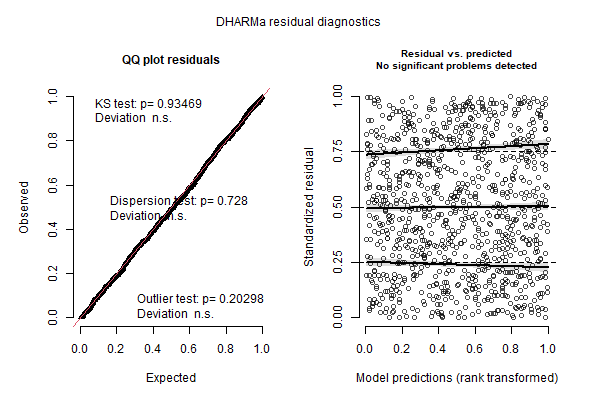


(d) Litter size


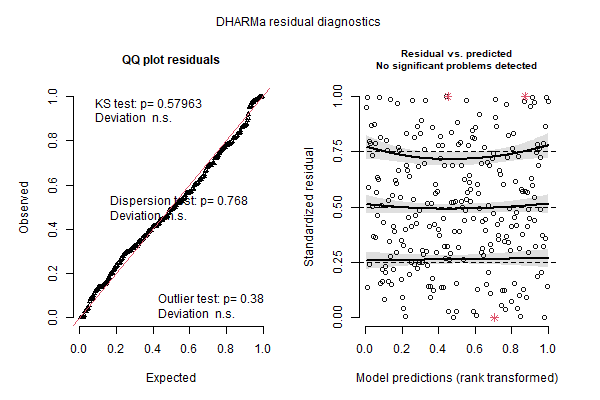


**Figure S3 continued.**

**Figure S4 (a-d).** QQ-plot (left) and plot of standardized residuals vs model predictions (right) simulated from the fitted models of Rattus rattus reproductive rates inside houses (Table S18). Red lines indicate quantile deviations detected.

(a) Female maturity (m1)


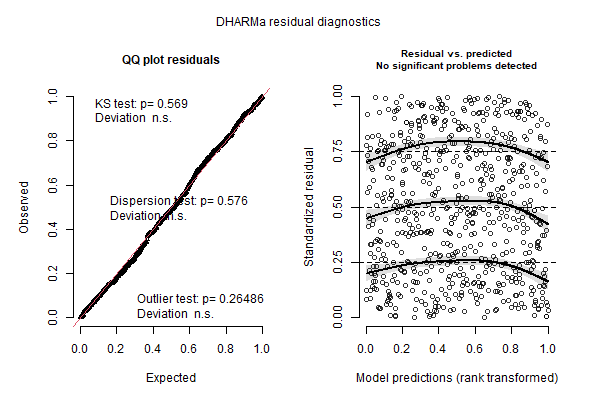


(b) Female maturity (m2)


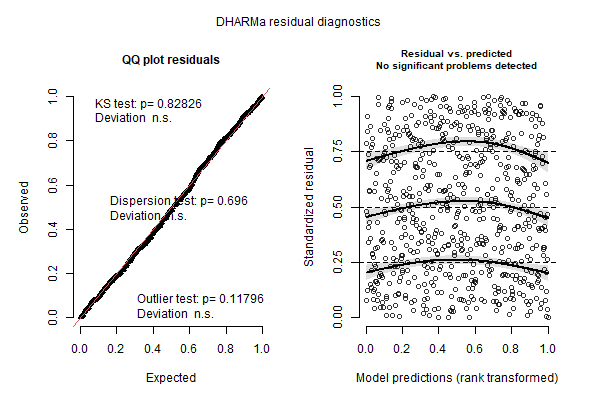


**Figure S4 continued.**

(c) Gestation rate


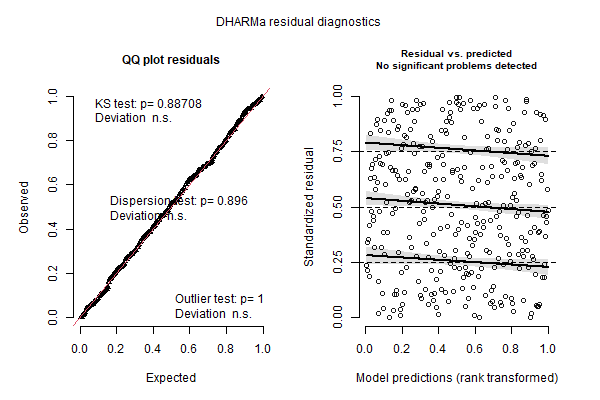


(d) Litter size


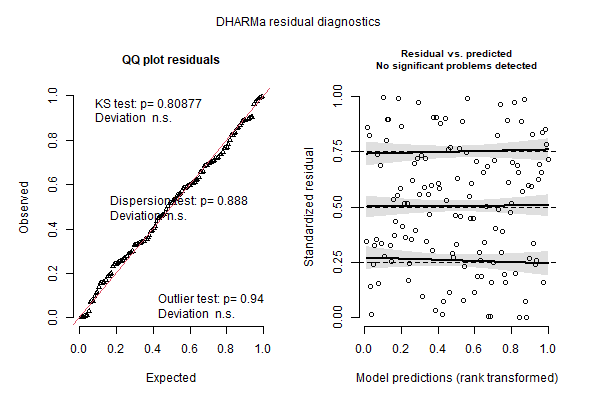

Supplement: Supplementary file 4 — Table S19–Table S28 AICc values and standardized regression coefficients from GLMM analysis of variables predicting litter size of pregnant Rattus rattus inside houses, where rainfall and population density were included as independent variables in global models. [file INZ2-19-66-s003.docx]
