# Supplementary material for: Reproductive ecology of the black rat (Rattus rattus) in Madagascar: the influence of density‐dependent and ‐independent effects
Source: Integr Zool. 2023 Jul 11;19(1):66–86. doi: 10.1111/1749-4877.12750 (PMC10952345; doi:10.1111/1749-4877.12750)
Supplement: Supplementary file 5 — Supporting Information [file INZ2-19-66-s004.docx]

**Supplementary Materials -** **Model selection and top model sets (rainfall and density)**

**Table S19** Evaluation of different rainfall characterisations for GLMM analysis of female Rattus rattus reproductive rates. Characterisations were based on 30-day rainfall (lag.n) and 90-day accumulated rainfall (Acc.lag.n) lagged by 0-3 months. Characterisations were assessed as additive effects and in interaction with season. Models also included an additive effect of head-body length and a random effect of site and mission nested within site. Models within the same row were compared using corrected Akaike’s Information Criterion (AICc). All models within 2 of the lowest AICc value were then evaluated in interaction with bioclimate (see **Table S2)** except if a model with an interaction was within 2 AICc of the equivalent additive model, in which case only the interaction effect was included in the global model (bold).

|  | 30-day rainfall (*Lag.n*) | | | | 90-day rainfall (*Acc.lag.n*) | | | |
| --- | --- | --- | --- | --- | --- | --- | --- | --- |
| Lag months: | 0 | 1 | 2 | 3 | 0 | 1 | 2 | 3 |
| *Outside of houses* | | | | | | | | |
| **Female maturity** | | | | | | | | |
| Interaction | 2512.0 | 2511.3 | 2512.2 | 2510.5 | 2509.8 | 2508.2 | **2505.8** | 2509.2 |
| Additive | 2515.7 | 2515.2 | 2515.7 | 2518.3 | 2518.1 | 2513.3 | 2518.6 | 2512.6 |
| **Gestation rate** | | | | | | | | |
| Interaction | 792.5 | 790.4 | 789.3 | 791.4 | 788.6 | 788.1 | 786.3 | **783.6** |
| Additive | 791.0 | 791.3 | 792.6 | 790.3 | 791.6 | 792.8 | 791.4 | 792.9 |
| **Litter size** | | | | | | | | |
| Interaction | **934.8** | 939.6 | 938.8 | 937.7 | 938.2 | 939.0 | **936.4** | 937.2 |
| Additive | **935.4** | **935.5** | **935.4** | **935.9** | **935.8** | **935.2** | **935.1** | **935.8** |
| *Inside houses* | | | | | | | | |
| **Female maturity** | | | | | | | | |
| Interaction | **723.7** | 726.0 | **724.7** | 727.9 | **723.3** | **723.3** | **724.9** | 731.5 |
| Additive | 731.5 | 730.8 | 727.7 | 729.4 | 730.4 | 726.6 | 729.3 | 731.5 |
| **Gestation rate** | | | | | | | | |
| Interaction | 434.9 | 435.2 | 436.2 | **430.1** | 437.2 | 435.0 | 434.7 | 432.2 |
| Additive | 433.2 | **431.2** | 432.4 | 432.1 | 434.4 | 433.6 | 434.6 | **430.3** |
| **Litter size** | | | | | | | | |
| Interaction | 546.5 | 544.8 | 545.6 | **541.5** | 546.1 | 543.7 | 544.6 | 542.4 |
| Additive | **542.2** | **540.3** | 542.29 | 540.8 | **541.5** | **542.2** | **542.0** | **540.3** |

**Table S20** Evaluation of different rainfall and bioclimate characterisations for GLMM analysis of female Rattus rattus reproductive rates. Rainfall characterisations were those identified as informative in a model with season (see **Table S1**). Models included an interaction between rainfall and bioclimate, an additive effect of head-body length, and a random effect of site and mission nested within site. Models were compared using corrected Akaike’s Information Criterion (AICc). Models within 2 of the lowest AICc value (bold) were included in global models. ^ⴕ^ Interaction models could not converge and so equivalent additive model was tested.

| Bioclimate characterisations | | | | |
| --- | --- | --- | --- | --- |
|  | *Bio.2* | *Bio.3* | *Bio.4* | *Bio.5* |
| *Outside of houses* | | | | |
| **Female maturity** | | | | |
| *Acc.lag.2* | **2530.5** | 2534.3 | **2531.3** | 2535.1 |
| **Gestation rate** | | | | |
| *Acc.lag.3* | 884.8 | 886.7 | **882.6** | **882.9** ^ⴕ^ |
| **Litter size** | | | | |
| *Lag.0* | 954.6 | 957.3 | 956.7 ^ⴕ^ | 957.5 ^ⴕ^ |
| *Lag.1* | 945.5 | 946.2 | 947.6 ^ⴕ^ | 947.2 ^ⴕ^ |
| *Lag.2* | 943.5 | 944.3 | 945.3 ^ⴕ^ | 945.6 ^ⴕ^ |
| *Lag.3* | 948.7 | 949.1 | 950.8 ^ⴕ^ | 951.8 ^ⴕ^ |
| *Acc.lag.0* | 946.0 | 946.5 | 948.2 ^ⴕ^ | 947.6 ^ⴕ^ |
| *Acc.lag.1* | 939.2 | **936.9** | 941.1 ^ⴕ^ | 941.0 ^ⴕ^ |
| *Acc.lag.2* | 944.4 | 944.0 | 946.2 ^ⴕ^ | 947.1 ^ⴕ^ |
| *Acc.lag.3* | 951.3 | 952.8 | 953.7 ^ⴕ^ | 954.8 ^ⴕ^ |
| *Inside houses* | | | | |
| **Female maturity** | | | | |
| *Lag.0* | 733.3 | 735.0 | 734.6 | 736.1 |
| *Lag.2* | 727.5 | 728.3 | 731.6 | 732.4 |
| *Acc.lag.0* | 730.1 | 731.1 | 732.5 | 733.4 |
| *Acc.lag.1* | **726.9** | **725.3** | 730.4 | 728.4 |
| *Acc.lag.2* | 731.5 | **727.0** | 734.9 | 730.6 |
| **Gestation rate** | | | | |
| *Lag.1* | 439.3 | 441.8 | 446.6 | 449.3 |
| *Lag.3* | **432.9** | 436.3 | 440.6 | 444.1 |
| *Acc.lag.3* | 435.6 | 438.0 | 443.1 | 445.7 |
| **Litter size** | | | | |
| *Lag.0* | 541.6 | 544.4 | 548.4 | 551.5 |
| *Lag.1* | **538.0** | 541.6 | 545.1 | 548.9 |
| *Lag.3* | **538.9** | 541.1 | 541.7 | 545.1 |
| *Acc.lag.0* | **539.1** | 542.3 | 545.6 | 549.1 |
| *Acc.lag.1* | **538.7** | 541.1 | 544.1 | 546.8 |
| *Acc.lag.2* | **538.9** | 540.2 | 544.6 | 546.2 |
| *Acc.lag.3* | **537.4** | 539.4 | 543.6 | 546.0 |

**Table S21** Evaluation of rat density characterisations for GLMM analysis of female Rattus rattus reproductive rates. Four density characterisations were considered: total and adult-only population density estimated at the site-level and at the house or trapline level. Each characterisation was assessed as an additive effect and in interaction with head-body length which was modelled as a continuous variable (Length.cont) and as a two-level categorical variable allowing reproductive rates to vary between large and small individuals (Length.150 grouped individuals above and below 150 mm). Models also included a random effect of site and mission nested within site. Models in the same row were compared using corrected Akaike’s Information Criterion (AICc). All models within 2 of the lowest AICc value were included in subsequent global models except if a model with an interaction was within 2 AICc of the equivalent additive model, in which case only the interaction effect was included in the global model (bold).

|  | Interaction | | | | Additive | | | |
| --- | --- | --- | --- | --- | --- | --- | --- | --- |
|  | Site | | Trapline/House | | Site | | Trapline/House | |
|  | Total | Adult | Total | Adult | Total | Adult | Total | Adult |
| *Outside of houses* | | | | | | | | |
| **Female maturity** | | | | | | | | |
| Length.cont | 2511.4 | 2507.1 | 2522.2 | **2495.3** | 2525.5 | 2528.4 | 2530.2 | 2531.0 |
| Length.150 | 2741.3 | 2738.7 | 2746.7 | 2736.9 | 2754.9 | 2759.5 | 2759.0 | 2765.8 |
| **Gestation rate** | | | | | | | | |
| Length.cont | 882.3 | 884.3 | 873.3 | 885.2 | 881.2 | 882.8 | 873.1 | 883.7 |
| Length.150 | 878.5 | 881.7 | **870.6** | 884.1 | 880.2 | 881.9 | **872.0** | 882.8 |
| **Litter size** | | | | | | | | |
| Length.cont | **955.5** | **954.7** | **955.2** | **955.9** | **953.7** | **953.7** | **953.6** | **953.8** |
| Length.150 | 964.4 | 963.5 | 964.0 | 965.3 | 963.8 | 964.0 | 963.6 | 963.9 |
| *Inside houses* | | | | | | | | |
| **Female maturity** | | | | | | | | |
| Length.cont | **721.4** | **721.3** | 734.9 | 732.5 | 725.6 | 725.1 | 733.0 | 731.9 |
| Length.150 | 784.7 | 784.4 | 794.5 | 789.6 | 868.2 | 874.6 | 875.8 | 866.2 |
| **Gestation rate** | | | | | | | | |
| Length.cont | **435.4** | 436.9 | 436.0 | 436.2 | **433.8** | 435.1 | **434.3** | **435.1** |
| Length.150 | 437.5 | 438.9 | 437.5 | 438.9 | 435.9 | 436.9 | 436.1 | 437.1 |
| **Litter size** | | | | | | | | |
| Length.cont | **543.8** | **543.7** | **544.5** | **545.4** | **542.7** | **543.2** | **542.4** | **543.2** |
| Length.150 | 551.0 | 551.7 | 551..3 | 552.0 | 549.4 | 549.7 | 549.1 | 549.9 |

**Table S22** Evaluation of rat density characterisations for GLMM analysis of female Rattus rattus reproductive rates. Four density characterisations were considered: total and adult-only population density estimated at the site-level and at the house/trapline level. Each characterisation was assessed as an additive effect and in interaction with season. Models also included an additive effect of head-body length (Length.cont) and a random effect of site and mission nested within site. Models in the same row were compared using corrected Akaike’s Information Criterion (AICc). All models within 2 of the lowest AICc value were included in subsequent global models except if a model with an interaction was within 2 AICc of the equivalent additive model, in which case only the interaction effect was included in the global model (bold). – Indicates model did not converge.

|  | Interaction | | | | Additive | | | |
| --- | --- | --- | --- | --- | --- | --- | --- | --- |
|  | Site | | Trapline/House | | Site | | Trapline/House | |
|  | Total | Adult | Total | Adult | Total | Adult | Total | Adult |
| *Outside of houses* | | | | | | | | |
| **Female maturity** | 2511.2 | 2513.7 | 2517.3 | 2516.3 | **2508.6** | **2510.7** | 2513.4 | 2513.1 |
| **Gestation rate** | 796.3 | **792.3** | **792.0** | 793.8 | **792.9** | **791.1** | **792.1** | **791.8** |
| **Litter size** | 939.4 | 938.8 | 940.0 | 939.7 | **935.8** | **935.9** | **935.8** | **935.8** |
| *Inside houses* | | | | | | | | |
| **Female maturity** | 729.9 | 729.3 | 732.3 | 730.7 | **726.7** | **726.5** | 731.4 | 730.5 |
| **Gestation rate** | 432.8 | 437.6 | **430.8** | 434.9 | **429.7** | 434.7 | 433.6 | 434.5 |
| **Litter size** | 546.2 | 546.2 | 546.5 | 546.4 | *–* | **541.6** | **542.0** | **542.3** |

**Table S23** AICc values and standardized regression coefficients from GLMM analysis of variables predicting maturity in female Rattus rattus outside of houses, where rainfall and population density were included as independent variables in global models. Models within the top model set are presented (m1-2) (i.e., models with an AICc within 2 of the lowest AICc but excluding those which include only one of either Season.sin or Season.cos). Model m2 was nested within model m1 and m1 was selected as the final model. ^ⴕ^Confidence intervals overlap zero. Bio.4 reference level = Arid. Within_village.vs.Outside_village reference level = Outside of village proximity.

|  | **m1** | **m2** |
| --- | --- | --- |
| **AICc** | 2482.3 | 2483.5 |
| **Log likelihood** | -1225.0 | -1228.7 |
| **β coefficients** |  |  |
| Intercept | -3.45 | -1.49 |
| Rainfall |  |  |
| *Acc.lag.2* | -0.71 ^ⴕ^ | -0.79 ^ⴕ^ |
| *Season.sin* | 1.73 | 1.86 |
| *Season.cos* | -0.76 | -0.81 |
| Site-level density (total) | -0.36 | -0.36 |
| *Within_village.vs.Outside_village* : Village proximity | 0.27 ^ⴕ^ | 0.21 ^ⴕ^ |
| *Length.cont* | 1.53 | 1.52 |
| *Acc.lag.2* x *Season.cos* | -0.4^ⴕ^ | -0.47 ^ⴕ^ |
| *Acc.lag.2* x *Season.sin* | 1.03 | 1.03 |
| *Acc.lag.2* x *Within_village.vs.Outside_village*: Village proximity | -0.49 | -0.47 |
| *Bio.4* : Temperate (all) | 1.96 | - |
| *Bio.4* : Tropical (rainforest) | 2.68 | - |
| *Bio.4* : Tropical (savannah) | 2.36 | - |
| Site-level density (total) x *Length.cont* | -0.3 | -0.3 |

**Table S24** AICc values and standardized regression coefficients from GLMM analysis of variables predicting gestation rates of sexually mature female Rattus rattus outside of houses, where rainfall and population density were included as independent variables in global models. Models within the top model set are presented (m1-4) (i.e., models with an AICc within 2 of the lowest AICc but excluding those which include only one of either Season.sin or Season.cos). Models m2-m4 were nested in model m1, which was selected as the final model. ^ⴕ^Confidence intervals overlap zero. Bio.4 reference level = Arid. Within_village.vs.Outside_village reference level = Outside of village proximity. Length.150 reference level = ≥150 mm.

|  | **m1** | **m2** | **m3** | **m4** |
| --- | --- | --- | --- | --- |
| **AICc** | 772.1 | 772.2 | 772.3 | 773.1 |
| **Log likelihood** | -367.7 | -369.8 | -368.8 | -372.3 |
| **β coefficients** |  |  |  |  |
| Intercept | -3.01^ⴕ^ | -3^ⴕ^ | -2.94^ⴕ^ | -2.71^ⴕ^ |
| *Season.sin* | 1.64 | 1.67 | 1.62 | 1.7 |
| *Season.cos* | 1.29 | 1.2 | 1.29 | 1.14 |
| Rainfall |  |  |  |  |
| *Acc.lag.3* | -3.83^ⴕ^ | -3.22^ⴕ^ | -3.65^ⴕ^ | -3.14^ⴕ^ |
| *Within_village.vs.Outside_village*: village proximity | -0.09^ⴕ^ | - | -0.05^ⴕ^ | - |
| *Bio.4* : Temperate (all) | 1.16^ⴕ^ | 1.14^ⴕ^ | 1.14^ⴕ^ | 0.94^ⴕ^ |
| *Bio.4* : Tropical (rainforest) | -50.89 | -53.45 | -51.21 | -54.37 |
| *Bio.4* : Tropical (savannah) | -0.75^ⴕ^ | -0.79^ⴕ^ | -0.63^ⴕ^ | -0.61^ⴕ^ |
| Site-level density (adult) | -0.24 | -0.24 | -0.22^ⴕ^ | -0.24 |
| *Acc.lag.3* x *Bio.4* |  |  |  |  |
| Temperate (all) | 3.67^ⴕ^ | 3.14^ⴕ^ | 3.52^ⴕ^ | 3.01^ⴕ^ |
| Tropical (rainforest) | -51.47 | -54.43 | -51.92 | -55.53 |
| Tropical (savannah) | 6.2^ⴕ^ | 5.87^ⴕ^ | 6.12^ⴕ^ | 5.78^ⴕ^ |
| *Season.sin* x *Acc.lag.3* | 0.63 | 0.59^ⴕ^ | 0.63 | 0.53^ⴕ^ |
| *Season.cos* x *Acc.lag.3* | 0.3^ⴕ^ | 0.25^ⴕ^ | 0.32^ⴕ^ | 0.32^ⴕ^ |
| *Within_village.vs.Outside_village* x *Acc.lag.3* | 0.59^ⴕ^ | - | 0.52^ⴕ^ | - |
| *Length.150* : <150 mm | 0.58^ⴕ^ | 0.44^ⴕ^ | - | - |

**Table S25** AICc values and standardized regression coefficients from GLMM analysis of variables predicting litter size of pregnant Rattus rattus outside of houses, where rainfall and population density were included as independent variables in global models. Models within the top model set are presented (m1-4) (i.e., models with an AICc within 2 of the lowest AICc but excluding those which include only one of either Season.sin or Season.cos). Models m1 and m4 were selected as the final models. ^ⴕ^Confidence intervals overlap zero. Within_village.vs.Outside_village reference level = Outside of village proximity.

|  | **m1** | **m2** | **m3** | **m4** |
| --- | --- | --- | --- | --- |
| **AICc** | 933.7 | 935.5 | 935.2 | 935.1 |
| **Log likelihood** | -459.6 | -459.4 | -459.3 | -461.4 |
| **β coefficients** |  |  |  |  |
| Intercept | 1.58 | 1.59 | 1.57 | 1.61 |
| *Season.sin* | 0.24 | 0.24 | 0.18^ⴕ^ | - |
| *Season.cos* | 0.01^ⴕ^ | 0.01^ⴕ^ | 0.03^ⴕ^ | - |
| Rainfall |  |  |  |  |
| *Acc.lag.1* | - | - | 0.04^ⴕ^ | 0.11 |
| *Within_village.vs.Outside_village* : Village proximity | - | -0.04^ⴕ^ | - | - |
| *Length.cont* | 0.11 | 0.11 | 0.11 | 0.1 |

**Table S26** AICc values and standardized regression coefficients from GLMM analysis of variables predicting maturity in female Rattus rattus inside houses, where rainfall and population density were included as independent variables in global models. Models within the top model set are presented (m1-4) (i.e., models with an AICc within 2 of the lowest AICc but excluding those which include only one of either Season.sin or Season.cos). Model m1 was selected as the final model. ^ⴕ^Confidence intervals overlap zero. Bio.2 reference level = non-temperate.

|  | **m1** | **m2** | **m3** | **m4** |
| --- | --- | --- | --- | --- |
| **AICc** | 717.3 | 719.3 | 717.4 | 718.4 |
| **Log likelihood** | -351.6 | -351.6 | -351.6 | -348.0 |
| **β coefficients** |  |  |  |  |
| Intercept | 0.4^ⴕ^ | 0.42^ⴕ^ | 0.39^ⴕ^ | -0.03^ⴕ^ |
| Rainfall |  |  |  |  |
| *Acc.lag.1* | 0.34 | 0.34 | 0.33 | -0.03^ⴕ^ |
| *Season.sin* | - | - | - | 0.15^ⴕ^ |
| *Season.cos* | - | - | - | -0.02^ⴕ^ |
| Site-level density |  |  |  |  |
| Total | -0.29^ⴕ^ | -0.28 | - | - |
| Adult-only | - | - | -0.59^ⴕ^ | -0.6^ⴕ^ |
| *Length.cont* | 0.83 | 0.83 | 0.89 | 0.91 |
| *Acc.lag.2* x *Season.cos* | - | - | - | -0.3^ⴕ^ |
| *Acc.lag.2* x *Season.sin* | - | - | - | 0.74 |
| *Bio.2* : Temperate | - | -0.06^ⴕ^ | - | - |
| Density x *Length.cont* | 0.27 | 0.27 | 0.53 | 0.5 |

**Table S27** AICc values and standardized regression coefficients from GLMM analysis of variables predicting gestation rates of sexually mature female Rattus rattus inside houses, where rainfall and population density were included as independent variables in global models. Models within the top model set are presented (m1-3) (i.e., models with an AICc within 2 of the lowest AICc but excluding those which include only one of either Season.sin or Season.cos). Models m1 and m3 were nested in model m2. Model m1 was selected as the final model. ^ⴕ^Confidence intervals overlap zero. Bio.2 reference level = non-temperate.

|  | **m1** | **m2** | **m3** |
| --- | --- | --- | --- |
| **AICc** | 426.3 | 426.6 | 426.8 |
| **Log likelihood** | -203.9 | -201.9 | -203.1 |
| **β coefficients** |  |  |  |
| Intercept | -0.43^ⴕ^ | -0.22^ⴕ^ | -0.14^ⴕ^ |
| Rainfall |  |  |  |
| *Lag.3* | 0.69^ⴕ^ | 0.78 | 0.86 |
| *Season.sin* | 0.04^ⴕ^ | -0.02^ⴕ^ | -0.08^ⴕ^ |
| *Season.cos* | 0.43^ⴕ^ | 0.44^ⴕ^ | 0.5^ⴕ^ |
| Site-level density (Total) | 0.39 | 0.4 | 0.42 |
| *Length.cont* | - | -0.2^ⴕ^ | - |
| *Lag.3* x *Season.cos* | -0.71^ⴕ^ | -0.58^ⴕ^ | -0.64^ⴕ^ |
| *Lag.3* x *Season.sin* | -0.45^ⴕ^ | -0.55^ⴕ^ | -0.57^ⴕ^ |
| *Bio.2* : Temperate | - | -0.25^ⴕ^ | -0.35^ⴕ^ |

**Table S28** AICc values and standardized regression coefficients from GLMM analysis of variables predicting litter size of pregnant Rattus rattus inside houses, where rainfall and population density were included as independent variables in global models. The top model set is presented (m1-12). Model m1 was selected as the final model. ^ⴕ^Confidence intervals overlap zero. Bio.2 reference level = non-temperate.

|  | **m1** | **m2** | **m3** | **m4** | **m5** | **m5** | **m6** |
| --- | --- | --- | --- | --- | --- | --- | --- |
| **AICc** | 536.0 | 536.7 | 537.2 | 537.5 | 537.7 | 537.8 | 537.9 |
| **Log likelihood** | -260.5 | -257.4 | -256.5 | -259.0 | -256.7 | -258.0 | -260.3 |
| **β coefficients** |  |  |  |  |  |  |  |
| Intercept | 1.61 | 1.61 | 1.6 | 1.63 | 1.62 | 1.64 | 1.64 |
| Rainfall |  |  |  |  |  |  |  |
| *Lag.1* | 0.06 | - | - | - | - | - | - |
| *Lag.3* | - | -0.17 | -0.16 | -0.08^ⴕ^ | -0.09^ⴕ^ | 0.01^ⴕ^ | -0.1 |
| *Acc.lag.0* | - | - | - | - | - | - | - |
| *Acc.lag.1* | - | - | - | - | - | - | - |
| *Acc.lag.3* | - | - | - | - | - | - | - |
| *Season.sin* | - | 0.22 | 0.21 | 0.16 | 0.2 | 0.14 | 0.19 |
| *Season.cos* | - | -0.11^ⴕ^ | -0.11^ⴕ^ | -0.08^ⴕ^ | -0.1^ⴕ^ | -0.07^ⴕ^ | -0.09^ⴕ^ |
| *Length.cont* | 0.08 |  | 0.05^ⴕ^ | 0.06^ⴕ^ |  | 0.06^ⴕ^ |  |
| Rain x *Season.cos* | - | 0.07^ⴕ^ | 0.05^ⴕ^ | - | 0.06^ⴕ^ | - | - |
| Rain x *Season.sin* | - | 0.11^ⴕ^ | 0.11^ⴕ^ | - | 0.1^ⴕ^ | - | - |
| *Bio.2* : Temperate | 0.12^ⴕ^ | 0.19 | 0.16 | 0.15 | 0.18 | 0.15 | 0.18 |
| Rain x *Bio.2* | - | - | - | - | -0.08^ⴕ^ | -0.1^ⴕ^ | - |

**Table S28 continued.**

|  | **m5** | **m6** | **m7** | **m8** | **m9** | **m10** | **m11** | **m12** |
| --- | --- | --- | --- | --- | --- | --- | --- | --- |
| **AICc** | 537.8 | 537.9 | 537.7 | 536.9 | 537.0 | 537.4 | 537.2 | 536.9 |
| **Log likelihood** | -258.0 | -260.3 | -262.5 | -258.7 | -257.5 | -260.1 | -261.1 | -261.0 |
| **β coefficients** |  |  |  |  |  |  |  |  |
| Intercept | 1.64 | 1.64 | 1.68 | 1.6 | 1.68 | 1.74 | 1.62 | 1.61 |
| Rainfall |  |  |  |  |  |  |  |  |
| *Lag.1* | - | - | - | - | - | - | - | - |
| *Lag.3* | 0.01^ⴕ^ | -0.1 | 0.06 | - | - | - | - | - |
| *Acc.lag.0* | - | - | - | - | - | - | - | 0.05^ⴕ^ |
| *Acc.lag.1* | - | - | - | - | - | - | 0.05^ⴕ^ | - |
| *Acc.lag.3* | - | - | - | -0.14^ⴕ^ | -0.01^ⴕ^ | 0.17 | - | - |
| *Season.sin* | 0.14 | 0.19 | - | 0.14 | 0.11 | - | - | - |
| *Season.cos* | -0.07^ⴕ^ | -0.09^ⴕ^ | - | -0.17^ⴕ^ | -0.15^ⴕ^ | - | - | - |
| *Length.cont* | 0.06^ⴕ^ |  | 0.1 | 0.07^ⴕ^ | 0.07^ⴕ^ | 0.08 | 0.09 | 0.08 |
| Rain x *Season.cos* | - | - | - | - | - | - | - | - |
| Rain x *Season.sin* | - | - | - | - | - | - | - | - |
| *Bio.2* : Temperate | 0.15 | 0.18 |  | 0.15 | 0.07^ⴕ^ | 0.02^ⴕ^ | 0.12^ⴕ^ | 0.13 |
| Rain x *Bio.2* | -0.1^ⴕ^ | - | - | - | -0.15^ⴕ^ | -0.22 | - | - |

(a) Female maturity


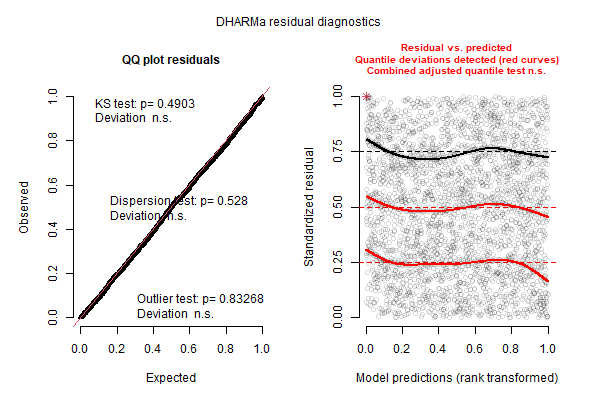


(b) Gestation rate


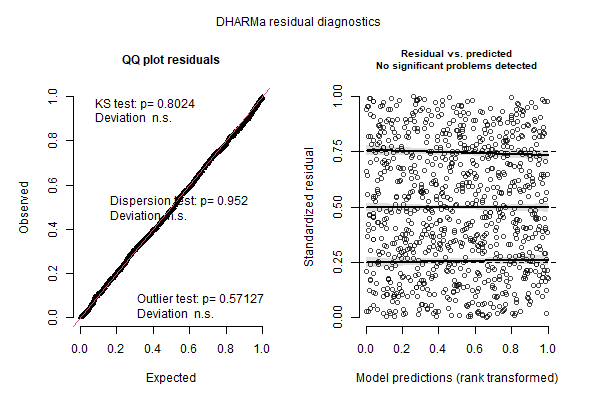


**Figure S5 (a-d).** QQ-plot (left) and plot of standardized residuals vs model predictions (right) simulated from the fitted models of Rattus rattus reproductive rates outside of houses (Table 3, main text). Red lines indicate quantile deviations detected.

(c) Litter size (m1)


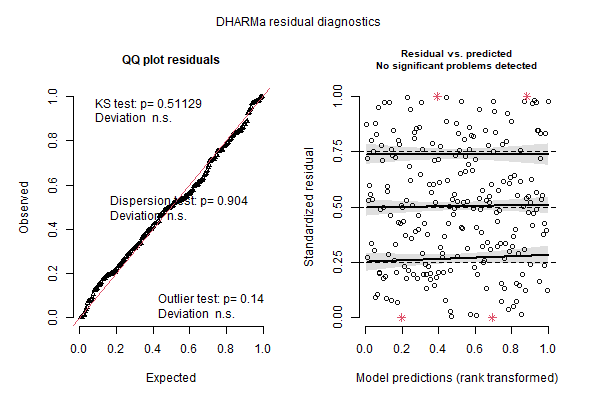


(d) Litter size (m2)


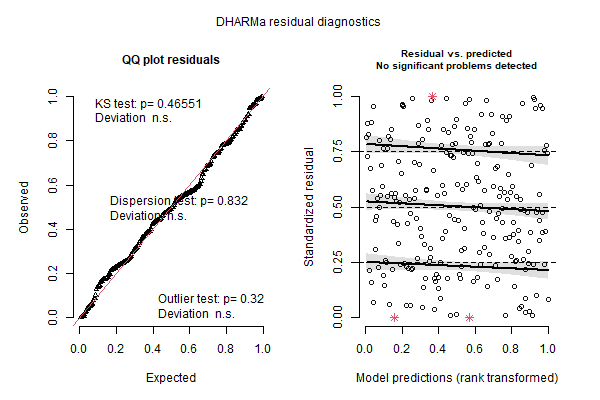


**Figure S5 continued.**

(a) Female maturity


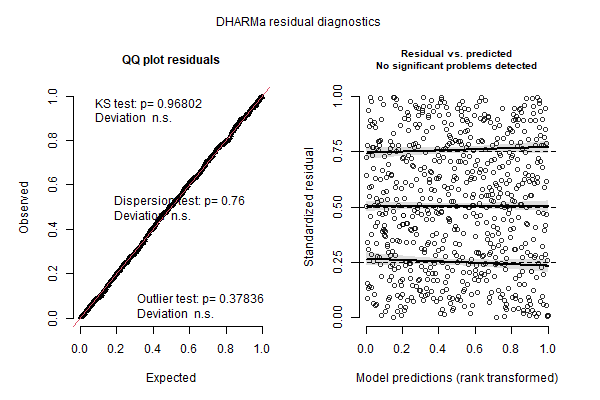


(b) Gestation rate


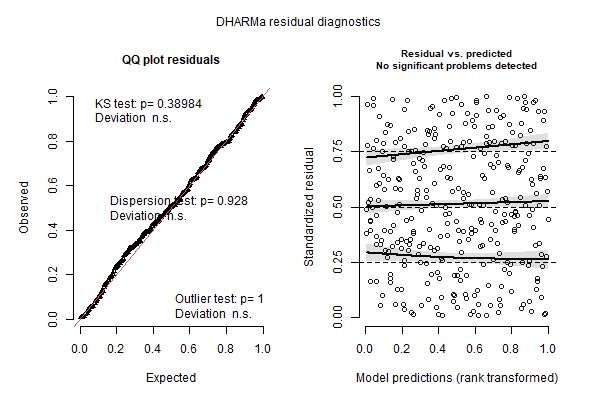


**Figure S6 (a-c).** QQ-plot (left) and plot of standardized residuals vs model predictions (right) simulated from the fitted models of Rattus rattus reproductive rates inside houses (Table 4, main text). Red lines indicate quantile deviations detected.

(c) Litter size


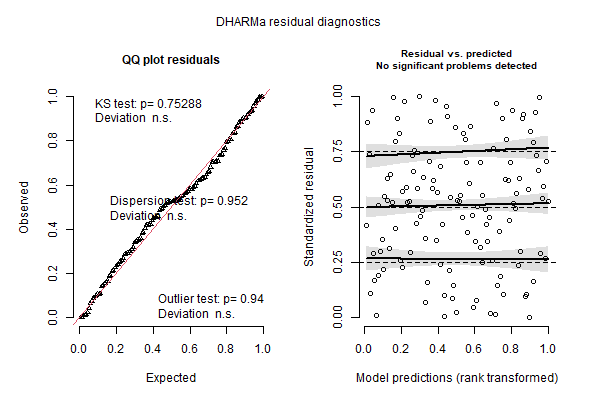


**Figure S6 continued.**
